# Pharmacological Blockade of Cannabinoid CB_1_ Receptors in Diet-Induced Obesity Regulates Mitochondrial Dihydrolipoamide Dehydrogenase in Muscle

**DOI:** 10.1371/journal.pone.0145244

**Published:** 2015-12-15

**Authors:** Sergio Arrabal, Miguel Angel Lucena, Miren Josune Canduela, Almudena Ramos-Uriarte, Patricia Rivera, Antonia Serrano, Francisco Javier Pavón, Juan Decara, Antonio Vargas, Elena Baixeras, Mercedes Martín-Rufián, Javier Márquez, Pedro Fernández-Llébrez, Baukje De Roos, Pedro Grandes, Fernando Rodríguez de Fonseca, Juan Suárez

**Affiliations:** 1 UGC Salud Mental, Instituto de Investigación Biomédica de Málaga (IBIMA), Universidad de Málaga-Hospital Universitario Regional de Málaga, Málaga, Spain; 2 CIBER OBN, Instituto de Salud Carlos III, Madrid, Spain; 3 Department of Neurosciences, University of the Basque Country UPV/EHU, Leioa, Spain; 4 ECAI de Proteómica, Instituto de Investigación Biomédica de Málaga (IBIMA), Universidad de Málaga, Málaga, Spain; 5 Departamento de Biología Molecular y Bioquímica, Instituto de Investigación Biomédica de Málaga (IBIMA), Universidad de Málaga, Málaga, Spain; 6 Departamento de Biología Celular, Genética y Fisiología, Instituto de Investigación Biomédica de Málaga (IBIMA), Universidad de Málaga, Málaga, Spain; 7 University of Aberdeen, Rowett Institute of Nutrition & Health, Aberdeen, United Kingdom; Laurentian University, CANADA

## Abstract

Cannabinoid CB_1_ receptors peripherally modulate energy metabolism. Here, we investigated the role of CB_1_ receptors in the expression of glucose/pyruvate/tricarboxylic acid (TCA) metabolism in rat abdominal muscle. Dihydrolipoamide dehydrogenase (DLD), a flavoprotein component (E3) of α-ketoacid dehydrogenase complexes with diaphorase activity in mitochondria, was specifically analyzed. After assessing the effectiveness of the CB_1_ receptor antagonist AM251 (3 mg kg^-1^, 14 days) on food intake and body weight, we could identified seven key enzymes from either glycolytic pathway or TCA cycle—regulated by both diet and CB_1_ receptor activity—through comprehensive proteomic approaches involving two-dimensional electrophoresis and MALDI-TOF/LC-ESI trap mass spectrometry. These enzymes were glucose 6-phosphate isomerase (GPI), triosephosphate isomerase (TPI), enolase (Eno3), lactate dehydrogenase (LDHa), glyoxalase-1 (Glo1) and the mitochondrial DLD, whose expressions were modified by AM251 in hypercaloric diet-induced obesity. Specifically, AM251 blocked high-carbohydrate diet (HCD)-induced expression of GPI, TPI, Eno3 and LDHa, suggesting a down-regulation of glucose/pyruvate/lactate pathways under glucose availability. AM251 reversed the HCD-inhibited expression of Glo1 and DLD in the muscle, and the DLD and CB_1_ receptor expression in the mitochondrial fraction. Interestingly, we identified the presence of CB_1_ receptors at the membrane of striate muscle mitochondria. DLD over-expression was confirmed in muscle of *CB*_*1*_^-/-^ mice. AM251 increased the pyruvate dehydrogenase and glutathione reductase activity in C_2_C_12_ myotubes, and the diaphorase/oxidative activity in the mitochondria fraction. These results indicated an up-regulation of methylglyoxal and TCA cycle activity. Findings suggest that CB_1_ receptors in muscle modulate glucose/pyruvate/lactate pathways and mitochondrial oxidative activity by targeting DLD.

## Introduction

The endocannabinoid signaling system (ECS) plays a fundamental role in the onset of obesity and metabolic disorders, implicating central and peripheral mechanisms predominantly via the activation of the cannabinoid CB_1_ receptors [1–4]. Obesity is associated with an increase in circulating endocannabinoid levels as a consequence of an altered expression of the endocannabinoid-metabolizing enzymes [5,6]. CB_1_ receptors are mainly expressed in the brain, but also in many peripheral organs involved in energy metabolism, including striate muscle and adipose tissue [7–9]. We now know that cannabinoids facilitates energy intake and, perhaps even more important, enhance energy storage into adipose tissue and reduce energy expenditure in muscle via lipid and glucose metabolism [5,10]. The endocannabinoid anandamide can modify β-oxidation pathways in the striate muscle, suggesting that CB_1_ receptor antagonism could be an important strategy in the regulation of energy expenditure needed to fight obesity [11]. Importantly, metabolic alterations related to obesity, due to an increased nutrient intake and/or a decreased fatty acid oxidation, are associated with insulin resistance, with direct effects on glucose uptake, energy expenditure and mitochondrial function in myocytes [12,13]. As a consequence, deficiency in the skeletal muscle activity produced by insulin resistance and obesity-linked metabolic dysfunction can be a major risk factor in the development of the so-called metabolic syndrome, leading to important diseases, such as type 2 diabetes mellitus or cardiovascular diseases [14,15].

CB_1_ receptor antagonists (e.g. Rimonabant, AM251) were developed as anti-obesity drugs, as they are able to antagonize hyperphagia, lead to weight loss and improve cardiometabolic risk profile and insulin resistance in genetic and dietary animal models of obesity [16–18]. However, SR141716A, which revealed metabolic benefits and body weight reduction in overweight and obese human subjects [19,20], induced psychiatric side effects, such as depression and anxiety, likely derived from a central CB_1_ receptor inverse agonist activity and leading to the withdrawal of the drug from the market [21,22]. These adverse effects highlighted the importance of limiting CB_1_ receptor antagonism to peripheral organs in order to reduce potential central risks and enhance peripheral energy balance [23]. However, information is lacking regarding the impact of CB_1_ receptors on the peripheral molecular mechanisms of glucose utilization and energy expenditure, which largely depend on muscle metabolism [8].

Here, we identified and evaluated changes in the expression profile of proteins involved in muscle metabolism, induced by CB_1_ receptor blockade in the presence or absence of diet-induced obesity (DIO). We firstly evaluated the most effective dose at which the CB_1_ receptor antagonist AM251 reduced food intake. After confirming that the repeated administration (14 days) of AM251 (3 mg kg^-1^) reduced food/caloric intake and body weight gain in rats fed with a high-fat diet (HFD) and a high-carbohydrate diet (HCD) for 10 weeks, we then analyzed both protein and gene expression of key enzymes involved in the regulation of the glucose/pyruvate/TCA pathways in the abdominal (rectus abdominis) striate muscle. Muscle dihydrolipoamide dehydrogenase was identified as a relevant mitochondrial enzyme of the TCA cycle regulated by CB_1_ receptors. This target was then corroborated in the striate muscle of *CB*_*1*_^-/-^ mice, and the mitochondria of striate muscle cell of HCD-fed rats. Finally, results were evaluated in conjunction with changes in the diaphorase/oxidative, pyruvate dehydrogenase and glutathione reductase activity in an *in vitro* model, using mytube-differentiated C_2_C_12_ cells and its mitochondria. These results were finally interpreted regarding the subcellular localization of the CB_1_ receptors in the striate muscle and its potential role in the mitochondrial oxidative metabolism.

## Materials and Methods

### Ethics statements

Experimental procedures with animals were carried out in strict accordance with the recommendations in the European Communities directive 2010/63/EU and Spanish legislation (Real Decreto 53/2013, BOE 34/11370-11421, 2013) regulating the care and use of laboratory animals. The protocol was approved by the Ethics Committee for Animal Experiments of the University of Malaga (Permit number: 2014/0003). All efforts were made to minimize animal suffering and to reduce the number of animals used.

### Animals

Ten-weeks-old male Wistar rats (Charles Rivers) and 7-weeks-old male *CB*_*1*_^-/-^ mice on a C57BL/6NCrl background [24] were individually housed in standardized conditions of animal facilities: water *ad libitum*, 20±2°C room temperature, 40±5% relative humidity and a 12-hours light/dark cycle (off at 8 pm) with dawn/dusk effect.

### Diet-induced obesity (DIO)

Rats were fed *ad libitum* for 12 weeks with three diets (*n* = 16): a standard diet (STD; Harlam), a high-fat diet (HFD, 60% fat diet; D12492, Research Diets Inc), or a high-carbohydrate diet (HCD, 70% carbohydrate diet; D12450B, Research Diets Inc). The HFD and HCD were used in order to induce obesity following previous studies [10]. See S1 Text for extended methodology.

### AM251 treatment

A dose-response study was firstly tested to select the most effective treatment for the repeated study. Rats (*n* = 8) fed with STD received one intraperitoneal (i.p.) injection of either vehicle (1 mL kg^-1^ of 10% Tocrisolve in saline) or CB_1_ receptor antagonist AM251 [N-(piperidin-1-yl)-5-(4-iodophenyl)-1-(2,4dichlorophenyl-4-methyl-1H-pyrazole-3-carboxamide] (Tocris; PubChem, CID:2125) at doses of 0.3, 1, 3 and 10 mg kg^-1^ of body weight. The cumulative food intake was measured over a time course of 30, 60, 120 and 240 minutes in rats previously food-deprived for 24 hours with *ad libitum* access to water. The minimal dose at which treatment showed a robust effect on food intake was selected for the repeated treatment experiment.

For repeated treatment, rats (*n* = 8) fed with STD and HFD for 10 weeks received a daily i.p. injection of vehicle or AM251 at a dose of 3 mg kg^-1^ over 14 days. Food intake and body weight were monitored every two days along feeding and treatment. We generated six experimental groups (*n* = 8): STD-vehicle, STD-AM251, HFD-vehicle, HFD-AM251, HCD-vehicle and HCD-AM251.

### Sample collection

Striate muscle from abdominal wall was dissected and frozen at -80°C until mRNA or protein analyses. Muscle samples were also fixed in 4% formaldehyde for 24 hours and embedded in paraffin for histology. Haematoxylin and eosin staining was used for the evaluation of muscle fiber size, structure and inflammatory state.

### Preparation of soluble protein fraction and two-dimensional (2D) electrophoresis

Abdominal muscle samples were placed on a homogenization buffer supplemented with a protease inhibitor cocktail (Roche Complete tablets). Then, 100 μl of 2.5 M sucrose was added and centrifuged at 6,400 *g* for 5 minutes, and then at 40,000 *g* for 45 minutes at 4°C. The supernatant, correspondent to the cytoplasm fraction of proteins, was measured using the Bradford assay and frozen at -20°C until the 2D electrophoresis analysis.

Triplicate 2D polyacrylamide gels were performed for each animal (*n* = 6) to minimize the effects of intra-assay variation. Isoelectric focusing (IPG) was performed with a Protean IEF cell (BioRad) using IPG gel strips. Protein extracts (30 μg) were added to a rehydration buffer (see S1 Text for extended methodology) and added to strips (rehydrated for 18 hours, 50 V and focused at 40 kV h^-1^, 20°C). The strips were incubated in two equilibration buffers containing 130 mM dithiothreitol and 135 mM iodoacetamide respectively. The 2-D was performed on Criterion XT Bis-Tris 12% polyacrylamide gels (80V, 20 minutes and 160V, 70 minutes, 20°C). The gels were fixed (40% ethanol, 10% acetic acid) for 16 hours, stained using a MS compatible method (Dodeca™ silver stain kit, Bio-Rad) and analyzed using PDQuest® Software v7.1 (Bio-Rad). Normalization of spots was made to compare the optic densities (OD) of the same spot (amount of protein) on other gels regarding the total density in gel image.

### MALDI-TOF and LC-ESI trap MS analyses

Spots with different OD were excised from the gels and trypsinized using the MassPrep Station (Waters, Micromass) protocol. Extracted peptides (1 μL) were applied to a 96×2 teflon MALDI target plate (Applied Biosystems), dried to ~50% of the original volume and placed in a α-cyano-4-hydroxycinnamic acid matrix solution. Gel plugs were also analyzed by nano-HPLC/ESI ion trap MS, using manual in-gel trypsin digestions of destained spots after reduction and alkylation with DTT and iodoacetamide, as described above (see S1 Text for extended methodology). MALDI-TOF mass spectrometry was performed using an Applied Biosystems Voyager-DE PRO in reflectron mode, as was described previously [25]. LC-ESI MS analysis was performed using an Agilent 1200 nano-HPLC system equipped with both PepMap100 C-18 trap and analytic columns [26].

### RNA isolation and qRT-PCR analysis

Total RNA was extracted from the abdominal muscle (∼100 mg) by using the Trizol method, as previously described [5]. Purified RNA (1 μg) and random hexamers were used to generate first strand cDNA using transcriptor reverse transcriptase. cDNA was used as a template for quantitative real-time PCR. The relative quantification was normalized to the expression of the housekeeping gene *Gapdh* and calculated by using the ΔΔCt method. Primers used for the qRT-PCR reaction were obtained based on TaqMan® Gene Expression Assays (Life Technologies) (Table 1).

**Table 1:** Primer References for TaqMan® Gene Expression Assays (ThermoFisher).

Gene Name	Assay ID	Amplicon Length
***Actb***	Rn00667869_m1	91
	Mm00607939_s1	115
***Gapdh***	Rn01775763_g1	175
	Mm99999915_g1	107
***Gusb***	Rn00566655_m1	63
	Mm01197698_m1	71
***Gpi***	Rn01475756_m1	85
	Mm01962484_u1	84
***Tpi1***	Rn03021693_g1	100
	Mm00833691_g1	197
***Eno3***	Rn01464911_m1	77
	Mm00468267_m1	54
***Pkm***	Rn00583975_m1	76
	Mm00834102_gH	182
***Dld***	Rn01648556_m1	68
	Mm00432831_m1	82
***Ldha***	Rn00820751_g1	85
	Mm01612132_g1	95
***Glo1***	Rn01429297_g1	84
	Mm00844954_s1	154
***Cox4i1***	Rn00665001_g1	72

*Actb*, β-actin; *Cox4i1*, cytochrome c oxidase subunit 4 isoform 1, mitocondrial; *Dld*, dihydrolipoamide dehydrogenase; *Eno3*, β-enolase; *Gapdh*, glyceraldehyde 3-phosphate dehydrogenase; *Glo1*, glyoxilase 1; *Gpi*, glucose-6-phosphate isomerase; *Gusb*, β-glucuronidase; *Ldha*, lactate dehydrogenase; *Pkm*, pyruvate kinase; *Tpi1*, triosephosphate isomerase 1.

### Total protein extraction and Western blot analysis

Protein extracts (75 μg) from mouse abdominal muscle were separated in gradient SDS-PAGE gels and electroblotted onto nitrocellulose membranes [10]. Specific proteins were detected by overnight incubation in the corresponding primary antibodies (Table 2). Then, a HRP-conjugated anti-rabbit IgG (H+L) or anti-mouse secondary antibodies (Promega) diluted 1:10,000 was added for 1 h at room temperature. After the enhanced chemiluminiscence detection (Santa Cruz) in an Autochemi-UVP Bioimaging System, bands were quantified by ImageJ software (Rasband, W.S., ImageJ, U.S., NIH, http://imagej.nih.gov/ij, 1997–2012).

**Table 2:** Antibodies Used for Protein Detection.

Protein	UniProt n°	Source Antibody	Antibody Dilution	Molecular Mass (kDa)
**β-actin**	P68134	Sigma (A5316)	1:2000	42
**GPI**	P06745	Santa Cruz (sc-33777)	1:500	63
**TPI**	P48500	Santa Cruz (sc-22031)	1:1000	30
**LDHa**	P04642	Cell signaling (2012S)	1:1000	37
**GLO1**	Q6P7Q4	Santa Cruz (sc-50731)	1:1000	24
**DLD**	O08749	Abcam (ab119422)	1:500–1:200	54
**CB**_**1**_	P47746	Abcam (ab23703)	1:200	52

See abbreviations in Table 1.

### Isolation of rat muscle mitochondria for protein (Western blot) analysis

The isolation of muscle mitochondria was realized as described previously [27] with modifications. Abdominal muscle was collected in isolation medium I (210 mM mannitol, 70 mM sucrose, 50 mM Trizma and 10 mM EDTA) and digested with trypsin (Gibco) at 0.5 mg g^-1^ for 30 minutes. Then, the reaction was stopped with a trypsin inhibitor and centrifuged at 1,000 *g* for 5 minutes. The supernatant was filtered and centrifuged at 7,000 *g* for 10 minutes. The obtained pellet was resuspended in an isolation medium II (225 mM mannitol, 75 mM sucrose, 10 mM Trizma and 0.1 mM EDTA) and centrifuged at 1,000 *g* for 5 minutes. The resulting supernatant was centrifuged at 7,000 *g* for 10 minutes. The obtained pellet (purified mitochondrial fraction) was resuspended in 50 μl of isolation medium II. Then, samples were homogenized by sonication and protein concentration was measured using the Bradford method with BSA as standard.

Western blotting of the mitochondrial protein extracts (30 μl) was performed as was described above. DLD and CB_1_ proteins were detected by overnight incubation in the corresponding primary antibodies diluted 1:200 (Table 2).

### Cell culture and treatment

C_2_C_12_ mouse C3H muscle myoblasts (cat. no. 91031101, Sigma-Aldrich) were cultured as previously described [28]. Proliferating C_2_C_12_ cells were propagated in DMEM (ThermoFisher) supplemented with 5% fetal bovine serum (FBS), 20 mM HEPES, 100 U ml^-1^ penicillin, 100 μg ml^-1^ streptomycin and 1% L-glutamine in a humidified atmosphere of 95% air/5% CO_2_ at 37°C. Differentiation was achieved upon exposure of proliferating C_2_C_12_ cells to a differentiation medium (DM) containing DMEM (25 mM glucose) supplemented with 2% horse serum, 20 mM HEPES, 100 U ml^-1^ penicillin, 100 μg ml^-1^ streptomycin and 1% L-glutamine for six days. To assess C_2_C_12_ myotube treatment, cells were first synchronized in fresh DM for 24 hours and then treated with the CB_1_ receptor agonist ACEA or the CB_1_ receptor antagonist AM251 at concentrations of 20, 50, 500, 10^3^ and/or 5∙10^3^ nM for 2 hours. After treatment, the cells were first cultured in fresh DM without serum for 2 hours and then incubated in 10 nM insulin for 10 minutes. Finally, 0.2 mg ml^-1^ Nitroblue Tetrazolium (NBT) was also added into the medium for a 3-hours incubation at 37°C in order to detect diaphorase/oxidative reaction at an optic density of 560 nm (VersaMax Absorbance Microplate Reader, Molecular Devices).

### Isolation of C_2_C_12_ myotube mitochondria

Mitochondrial extraction from differentiated C_2_C_12_ cells was realized as described previously [29] with modifications. Cells were trypsinised and centrifuged at 1,000 *g* for 10 minutes. The pellet was resuspended in a buffer I containing 210 mM mannitol, 70 mM sucrose, 5 mM HEPES, 1 mM EDTA and 0.5% BSA supplemented with 0.5 mM Tx100. After 15 minutes, permeabilization was verified using trypan blue. Samples were centrifuged at 625 *g* for 5 minutes and the pellet was resuspended in 10 ml of buffer I and homogenized with 60 gentle strikes in a glass potter. Membrane disruption was verified under the microscope, and cells were centrifuged at 625 *g* for 5 minutes. The supernatant was centrifuged at 10,000 *g* for 20 minutes, and the pellet (purified mitochondrial fraction) was resuspended in 100 μl of a respiration buffer (75 mM mannitol, 25 mM sucrose, 100 mM KCl, 10 mM Tris-HCl pH 7.4, 50 μM EDTA and 10 mM sodium pyruvate). Protein concentration was measured using the Bradford method with BSA as standard. Approximately, 1.1 mg of mitochondrial proteins per 10^6^ cells was recovered.

### Diaphorase activity measurement of C_2_C_12_ mitochondrial fraction

Mitochondrial samples at a final protein concentration of 0.2 mg ml^-1^ were incubated in respiration buffer for 10 minutes at 37°C. Samples were first treated with AM251 or ACEA at 20, 50, 500, 10^3^ and 5∙10^3^ nM for 30 minutes. Then, NBT were added for an overnight incubation at 37°C in order to detect diaphorase/oxidative reaction. Finally, samples were homogenized by sonication and measured at an optic density of 560 nm.

### Measurement of pyruvate dehydrogenase and glutathione reductase activity in C_2_C_12_ myotubes

Myotubes were first synchronized in fresh DM for 24 hours and then treated with ACEA at 1 μM or AM251 at 50 nM for 2 hours. Then, the cells were first cultured in fresh DM without serum for 2 hours and then incubated in 10 nM insulin for 10 minutes. Finally, the cells were processed following the protocols described in the respective commercial assay kits in order to kinetically detect pyruvate dehydrogenase reaction (cat. no. MAK183, Sigma-Aldrich), glutathione reductase reaction (OxiSelect^TM^ GR, cat. no. STA-812, Cell Biolabs, Inc.) and total glutathione content (OxiSelect^TM^ GSSG/GSH, cat. no. STA-312, Cell Biolabs, Inc.) at an optic density of 450, 405 and 405 nm respectively. One unit of pyruvate dehydrogenase is the amount of enzyme that generates 1 μmole of NADH per minute at pH 7.5 at 37°C. Pyruvate dehydrogenase activity was reported as nmole/min/mL relative to control group. Glutathione content was kinetically determined from the production rate of reduced glutathione (GSH) from oxidized glutathione (GSSG) for 12 minutes in presence of glutathione reductase and NADPH. Glutathione reductase activity was reported as mU/min/mL in presence of GSSG and NADPH.

### Electron microscopy

A preembedding silver-intensified immunogold method was used for the localization of CB_1_ protein as was described [30]. Fifty μm-thick-sections of muscular tissues were first incubated in primary goat CB_1_ polyclonal antibodies (2μg ml^-1^; CB1-Go-Af450-1; Frontier Science Co. Ltd) and then in a secondary 1.4 nm gold-labeled rabbit anti-goat IgG (Fab' fragment, 1:100, Nanoprobes Inc.). Gold particles were silver-intensified with a HQ Silver kit (Nanoprobes Inc.). Osmicated, plastic-embedded flat in Epon 812. 65 nm ultrathin sections were stained with uranyl acetate and examined in a Philips EM208S electron microscope.

### Statistical analysis

Data were expressed as the mean ± standard error of the mean (s.e.m.) of 6–8 determinations per experimental group. Statistical analysis were performed using one or two-way ANOVA with diet (STD, HFD and HCD) and treatment (vehicle and AM251) factors, followed by Bonferroni *post-hoc* test for multiple comparisons, or one-tailed Student’s *t*-test with the *Welch* correction. A *P*-value below 0.05 was considered statistically significant.

## Results

### AM251 effectiveness on food intake and body weight in DIO rats

**Fig 1:**
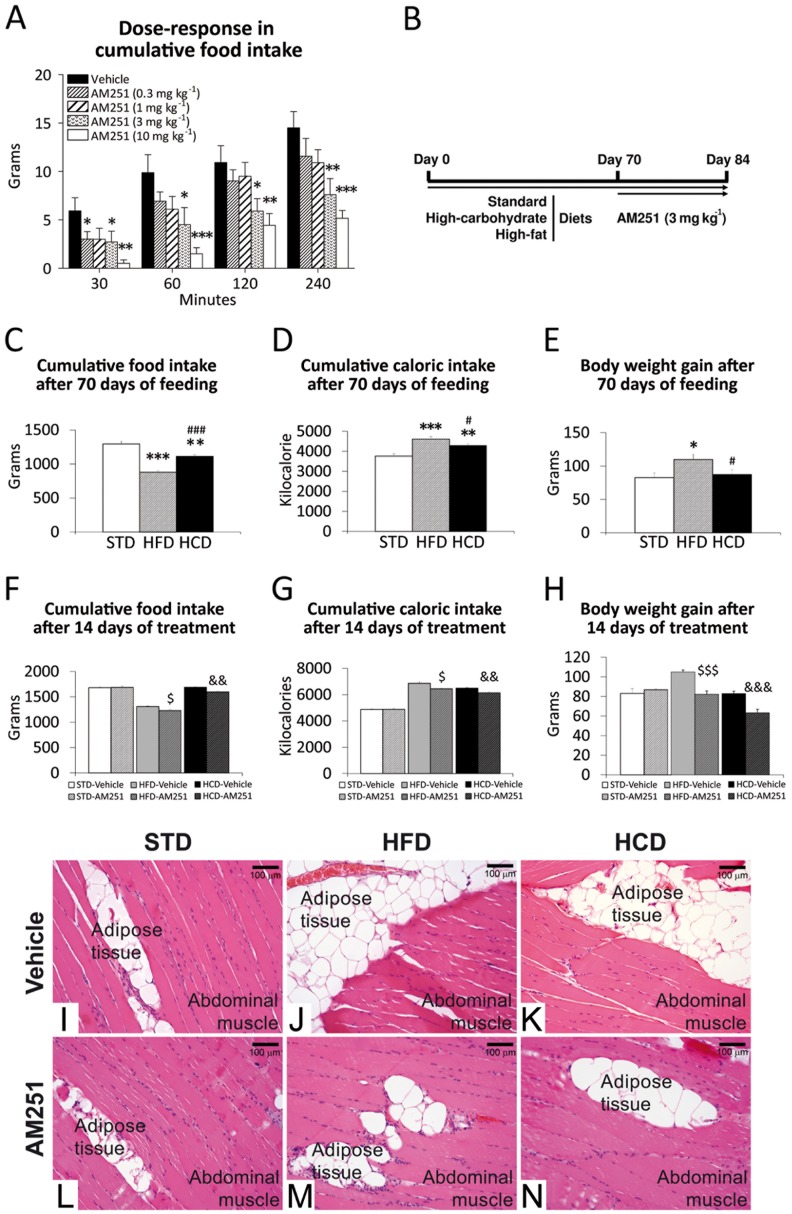
AM251 effectiveness on food intake and body weight in DIO rats. Dose-response of AM251 (0.3, 1, 3, 10 mg kg^-1^) in cumulative food intake (**A**) and experimental design (**B**) for cumulative food/caloric intake and body weight gain (**C**-**H**) during STD, HFD and HCD feeding for 84 days and repeated AM251 administration (3 mg kg^-1^) for 14 days. Bonferroni’s test (*n* = 8): *P<0.05, **P<0.01, ***P<0.001 *vs*. STD; ^#^ *P*<0.05, ^###^ *P*<0.001 *vs*. HFD; ^$^ *P*<0.05, ^$ $ $^ *P*<0.001 *vs*. HFD-vehicle; ^&&^ *P*<0.01, ^&&&^ *P*<0.001 *vs*. HCD-vehicle. Haematoxylin-eosin staining in the abdominal muscle of the rats fed with STD, HFD and HCD for 84 days and repeatedly treated with AM251 (3 mg kg^-1^) for 14 days (**I**-**N**).

AM251 administered at 3 and 10 mg kg^-1^ were the most effective doses on reducing cumulative food intake over 240 minutes of STD feeding in rats that had been previously food-deprived for 24 hours feeding (Fig 1A). So, the minimal dose of 3 mg kg^-1^ was selected for the repeated-treatment experiment (Fig 1B). After 70 days of HFD and HCD feeding to induce obesity (Fig 1C–1E), AM251 repeatedly administered at 3 mg kg^-1^ for 14 days produced significant reductions in cumulative food/caloric intake and body weight gain in a hypercaloric diet-independent manner (Fig 1F–1H). Haematoxylin and eosin staining in the abdominal muscle showed an apparent increase in the amount of adipose tissue located between the muscle fibers of the rats fed with HFD and HCD (Fig 1I–1K). However, the extension of adipose tissue located between the muscle fibers seemed to be reduced after the repeated administration of AM251 (Fig 1L–1N).

### Protein identification and characterization in muscle of DIO rats treated with AM251

**Fig 2:**
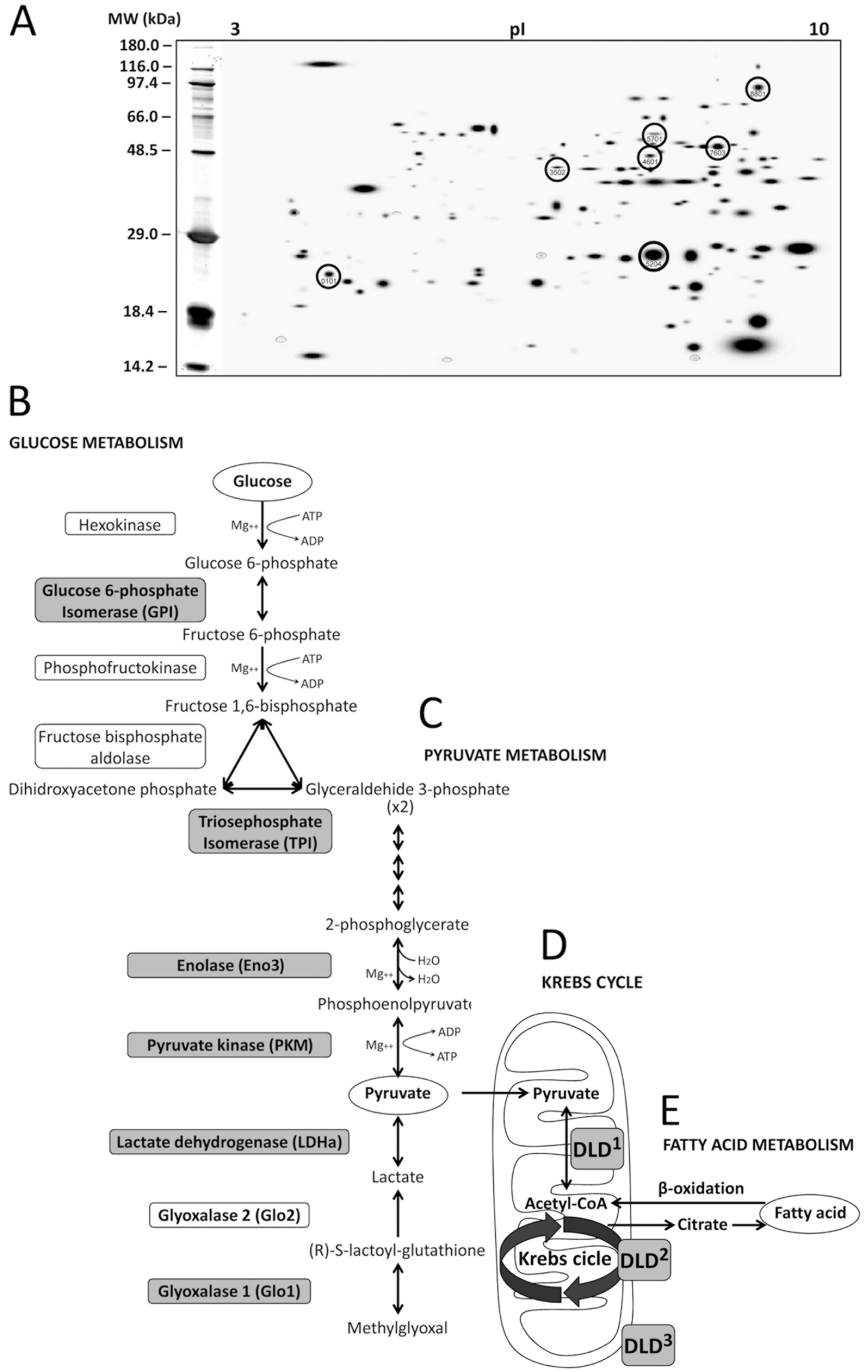
Protein identification and characterization in muscle of DIO rats treated with AM251. Seven proteins in the abdominal muscle of STD, HFD and HCD-fed rats treated with AM251 were identified by using 2-dimensional (2D) electrophoresis and MALDI-TOF/LC-ESI trap mass spectrometry. **A)** A representative 2D polyacrylamide gel showing the selected spots. **B-D**) Scheme of the glucose and pyruvate pathways and Krebs cycle showing the step reaction of the seven enzymes identified. **E**) Fatty acid metabolism. Dihydrolipoamide dehydrogenase (DLD) is an E3 component with dehydrogenase/diaphorase activity of the mitochondrial pyruvate^1^/α-ketoglutarate^2^/branched-chain α-ketoacid^3^ dehydrogenase complexes.

Through comprehensive proteomic approaches involving 2D electrophoresis in the rat muscle of the six experimental groups (STD-vehicle, STD-AM251, HFD-vehicle, HFD-AM251, HCD-vehicle and HCD-AM251), we selected 7 spots from up to 300 spots analyzed in each 2D gel, whose average of optic densities (protein content) significantly changed when the experimental groups were compared (Fig 2A). Each spot represented a single protein showing specific electrochemical properties and molecular weight. MALDI-TOF and LC-ESI MS analyses identified the same protein hits for the 7 spots: Mascot score, number of peptides, peptide coverage, molecular weight and isoelectric point, among other parameters, which are highly associated to the characteristics and the identity of 7 known proteins (Table 3). The 7 proteins identified in the abdominal muscle were glucose-6-phosphate isomerase (GPI), triosephosphate isomerase (TPI), beta-beta muscle specific enolase or enolase-3 (Eno3), pyruvate kinase isozyme M1 (PKM1), lactate dehydrogenase A-chain (LDHa), glyoxalase-1 (Glo1), and dihydrolipoamide dehydrogenase (DLD). These 7 proteins are key regulatory enzymes of the glucose, pyruvate and lactate metabolism as well as branched-chain amino acids of the tricarboxylic acid (Krebs) cycle in the striate muscle (Fig 2B–2E).

**Table 3:** Proteins Identified using 2D Electrophoresis and MALDI-TOF/LC-ESI MS Analyses.

ID No.	Score	Pept	COV	MW/pI	Protein Identified
**7603 Q6P6V0**	957	15	33	63.0/7.4	Glucose-6-phosphate isomerase (GPI)
**5204 P48500**	1169	13	64	27.3/6.5	Triosephosphate isomerase (TPI)
**3502 P15429**	114	6	16	47.0/7.1	Beta-enolase (Eno3 or MSE)
**5701 P11980**	76	6	11	58.3/6.6	Pyruvate kinase isozyme M1 (PKM1)
**8801 P04642**	319	12	37	36.7/8.4	Lactate dehydrogenase A chain (LDHa)
**0101 Q6P7Q4**	50	3	10	21.0/5.1	Glyoxalase 1 (Glo1)
**4601 Q6P6R2**	129	4	11	54.6/8.0	Dihydrolipoamide dehydrogenase (DLD)

ID, Identification number of the spot; Score, Mascot score from the MOWSE algorithm for peptide mass fingerprinting; Pept, number of peptides indentified; COV, peptide coverage over the protein; MW, molecular weight; pI, isoelectric point. For each hit, the higher Mascot score obtained from the two MS approaches was selected.

### AM251 regulates the protein expressions of glucose/pyruvate-metabolizing enzymes in the abdominal muscle of HCD-fed rats

**Fig 3:**
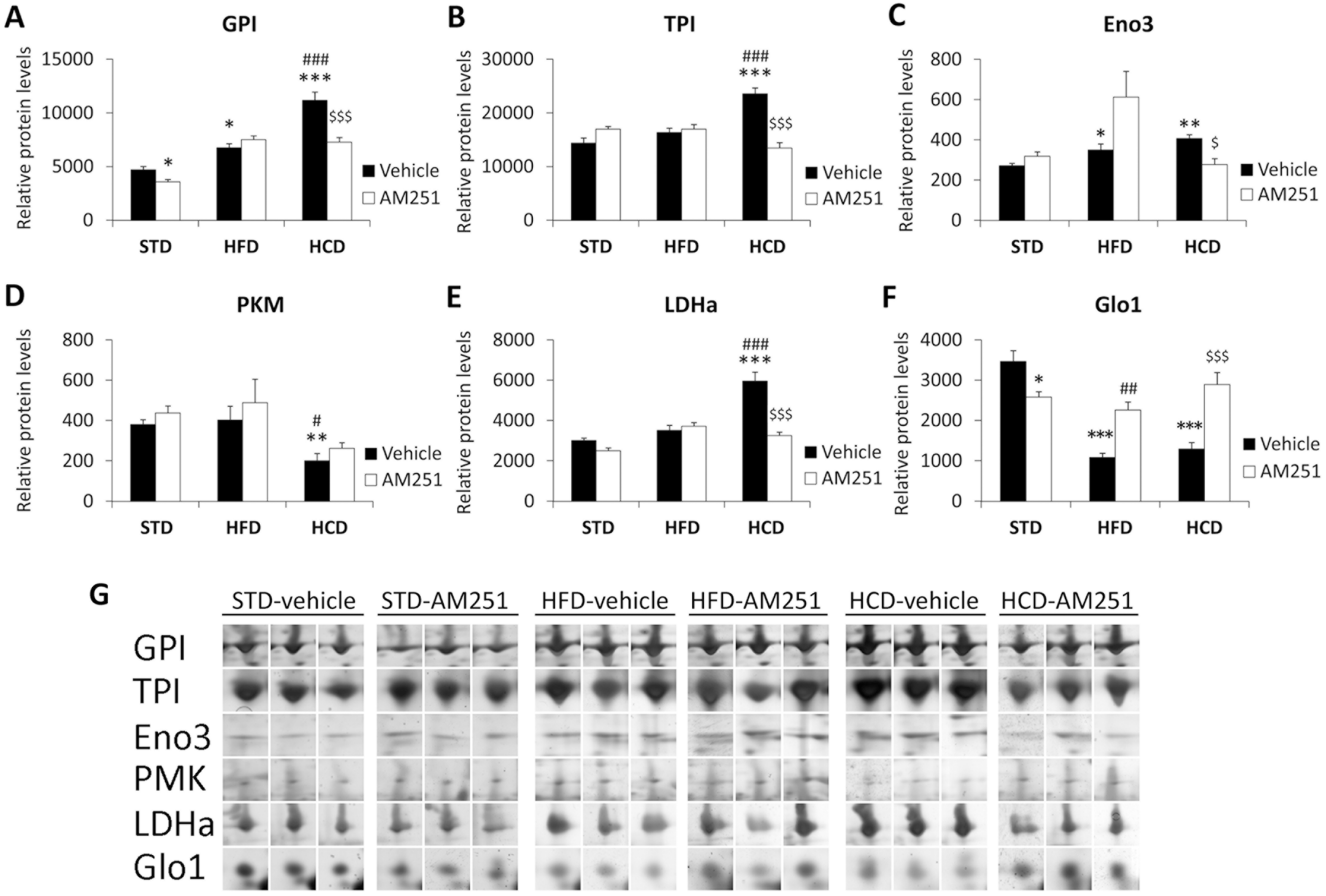
AM251 regulates the protein expressions of glucose/pyruvate-metabolizing enzymes. AM251 effects on the protein expressions of GPI, TPI, Eno3, PKM1, LDHa and Glo1 in the abdominal muscle of STD, HFD and HCD-fed rats (**A-F**). Representative 2D polyacrylamide gels showing the intensity of the identified spots (**G**). Bonferroni’s test (*n* = 6): *P<0.05, **P<0.01, ***P<0.001 *vs*. STD-vehicle; ^#^ *P*<0.05, ^##^ *P*<0.01, ^###^ *P*<0.001 *vs*. HFD-vehicle; ^$^ *P*<0.05, ^$ $ $^ *P*<0.001 *vs*. HCD-vehicle.

The expression of most proteins identified in muscle was mainly affected by the HCD and the AM251 treatment (Fig 3). Two-way ANOVA showed a diet effect on the protein expressions of GPI, TPI, LDHa and Glo1 (GPI/LDHa/Glo1: *F*_1,30_>13.88, *P*<0.001; TPI: *F*_1,30_ = 9.83, *P*<0.01). A treatment effect on the expressions of all proteins identified was observed (GPI/LDHa/Glo1: *F*_2,30_>22.28, *P*<0.0001; Eno3/PKM1: *F*_2,30_>5.43, *P*<0.01; TPI: *F*_2,30_ = 5.26, *P* = 0.011). Interactions between diet and treatment were detected for the protein expressions of GPI, TPI, Eno3, LDHa and Glo1 (GPI/TPI/LDHa/Glo1: *F*_2,30_>13.15, *P*<0.0001; Eno3: *F*_2,30_ = 5.59, *P*<0.01), indicating that the repeated AM251 administration differentially modified their protein expression in a diet-dependent manner.

Regarding Bonferroni test (Fig 3), we observed an increased GPI and Eno3 expressions in HFD-fed rats as well as a higher significance of the increased GPI, TPI, Eno3 and LDHa expressions in HCD-fed rats (*^/^**^/^***P<0.05/0.01/0.001) (Fig 3A–3C and 3E). Increased GPI, TPI and LDHa expressions were also observed in HCD-fed rats compared with HFD ones (^###^ *P*<0.001) (Fig 3A, 3B and 3E). Thus, these glucose/pyruvate metabolism enzymes were up-regulated as a consequence of a hypercaloric carbohydrate diet. In contrast, we detected a decreased Glo1 expression in HFD-fed rats as well PKM1 and Glo1 in HCD-fed rats (**^/^***P<0.01/0.001) (Fig 3D and 3F). Additionally, decreased PKM1 expression was also detected in HCD-fed rats compared with HFD ones (^#^ *P*<0.05) (Fig 3D). Thus, methylglyoxal pathway key enzymes were down-regulated after a hypercaloric carbohydrate diet.

AM251 decreased the GPI and Glo1 expressions in STD-fed rats (*P<0.05) (Fig 3A and 3F). AM251 also decreased the GPI, TPI, Eno3 and LHDa expressions specifically in HCD-fed rats (^$/$ $ $^ *P*<0.05/0.001) (Fig 3A–3C and 3E). Thus, the expressions of these glucose/pyruvate metabolism enzymes were specifically blocked after AM251 treatment in a highly-carbohydrate context. In contrast, AM251 increased the Glo1 expression in HFD (^##^ *P*<0.01) and HCD (^$ $ $^ *P*<0.001) (Fig 3F).

### AM251 regulates the gene expressions of glucose/pyruvate-metabolizing enzymes in the abdominal muscle of HCD-fed rats

**Fig 4:**
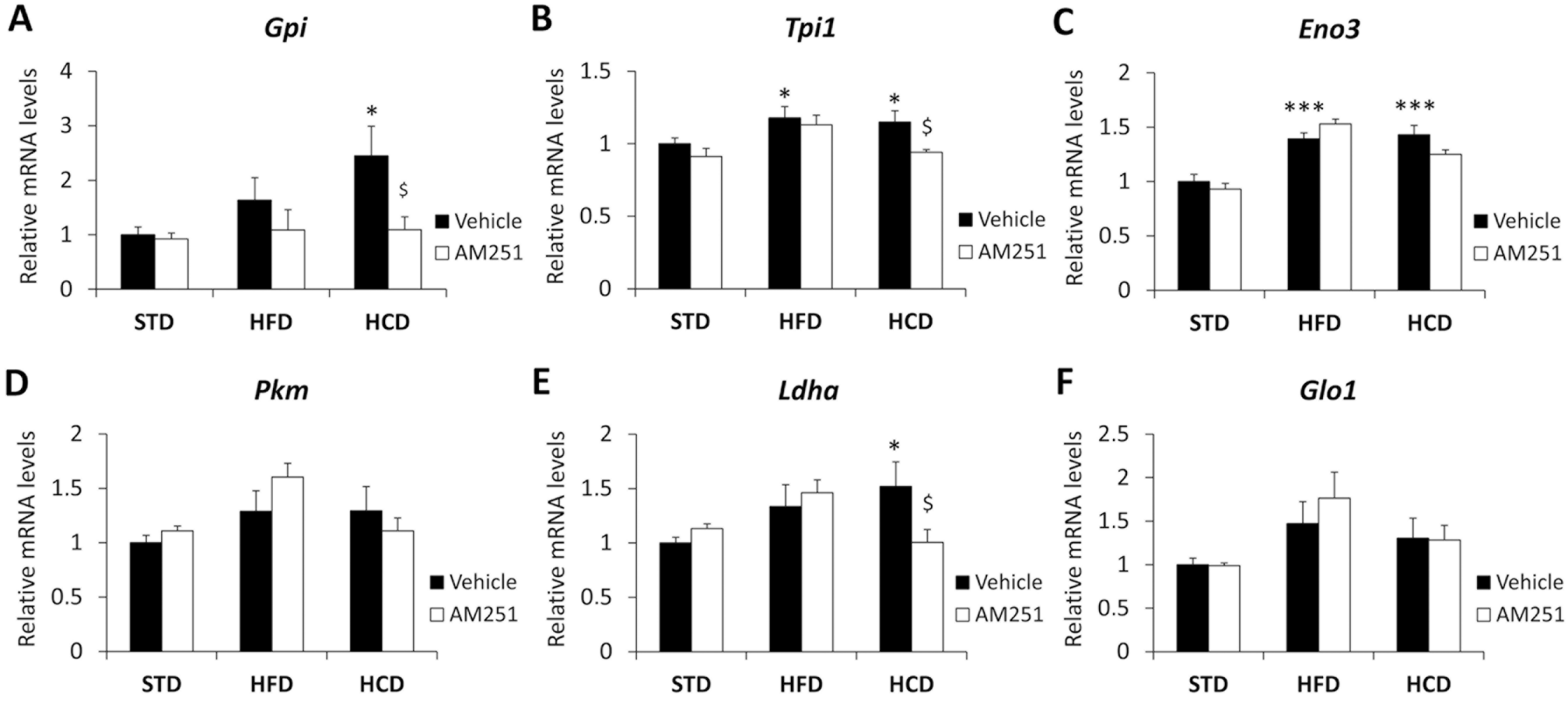
AM251 regulates the gene expressions of glucose/pyruvate-metabolizing enzymes. AM251 effects on the gene expressions of *Gpi*, *Tpi1*, *Eno3*, *Pkm*, *Ldha* and *Glo1* in the abdominal muscle of STD, HFD and HCD-fed rats. Bonferroni’s test (*n* = 8): *P<0.05, ***P<0.001 *vs*. STD-vehicle; ^$^ *P*<0.05 *vs*. HCD-vehicle.

Two-way ANOVA only showed a diet effect on the *Gpi* and *Tpi* expressions (*F*_1,38_>5.12, *P*<0.027) (Fig 4). A treatment effect on the *Tpi*, *Eno3*, *Pkm* and *Glo1* expressions were also observed (*Tpi*/*Pkm/Glo1*: *F*_2,37_>3.69, *P*<0.034; *Eno3*: *F*_2,39_ = 33.02, *P*<0.0001). Interaction was only detected for the *Ldha* expression (*F*_2,39_ = 3.44, *P* = 0.041), indicating that AM251 treatment differentially modified its gene expression of in a diet-dependent manner.

Regarding Bonferroni test (Fig 4), we observed an increased *Tpi* and *Eno3* expressions in HFD-fed rats as well as *Gpi*, *Tpi*, *Eno3* and *Ldha* in HCD-fed rats (*^/^***P<0.05/0.001) (Fig 4A–4C and 4E). AM251 only decreased the *Gpi*, *Tpi* and *Ldha* expressions in HCD-fed rats (^$^ *P*<0.05) (Fig 4A, 4B and 4E).

### The expression of the metabolic enzymes in the abdominal muscle of *CB*_*1*_^-/-^ mice was altered

**Fig 5:**
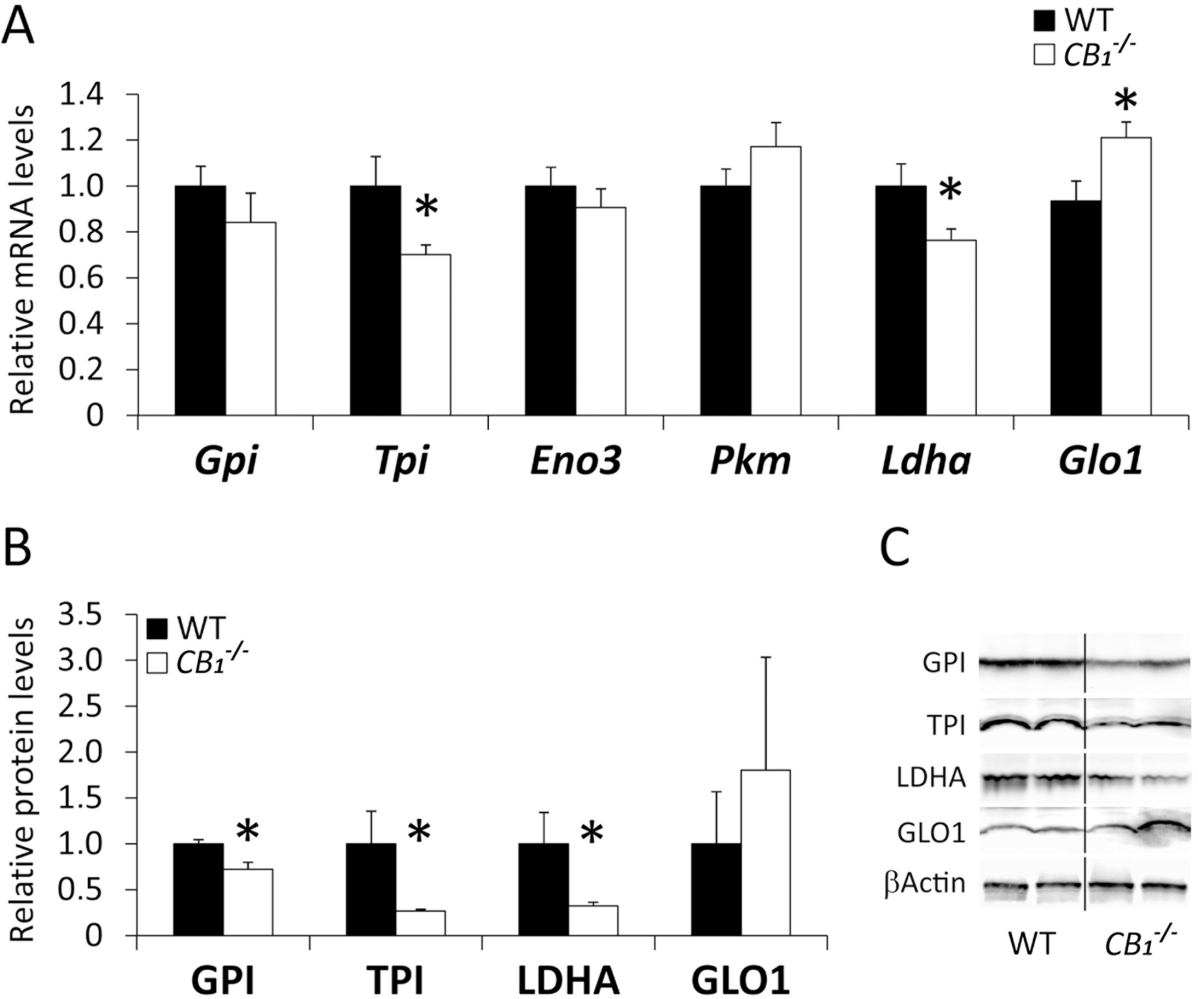
Expression of the metabolic enzymes in the abdominal muscle of *CB*_*1*_^-/-^ mice. Gene (**A**) and protein (**B**) expressions of GPI, TPI, Eno3, PKM1, LDHa and Glo1 in the abdominal muscle of wild-type and *CB*_*1*_^-/-^ mice. **C)** Representative immunoblots. Student’s *t* test (*n* = 6): *P<0.05 *vs*. WT.

To assess the results obtained in the AM251-treated DIO rats, the muscles of *CB*_*1*_^-/-^ mice were also analyzed by qRT-PCR and Western immunoblotting (Fig 5). *CB*_*1*_^-/-^ mice showed a decreased gene expression of *Tpi1* and *Ldha* (*P<0.05) (Fig 5A), which were confirmed regarding their protein expression (*P<0.05) (Fig 5B and 5C). We also observed an increased gene expression of *Glo1* (*P<0.05) (Fig 5A) and a decreased protein expression of GPI in *CB*_*1*_^-/-^ mice (*P<0.05) (Fig 5B).

### AM251 regulates the expression of DLD and Cox4i1 in the abdominal muscle of HCD-fed rats

**Fig 6:**
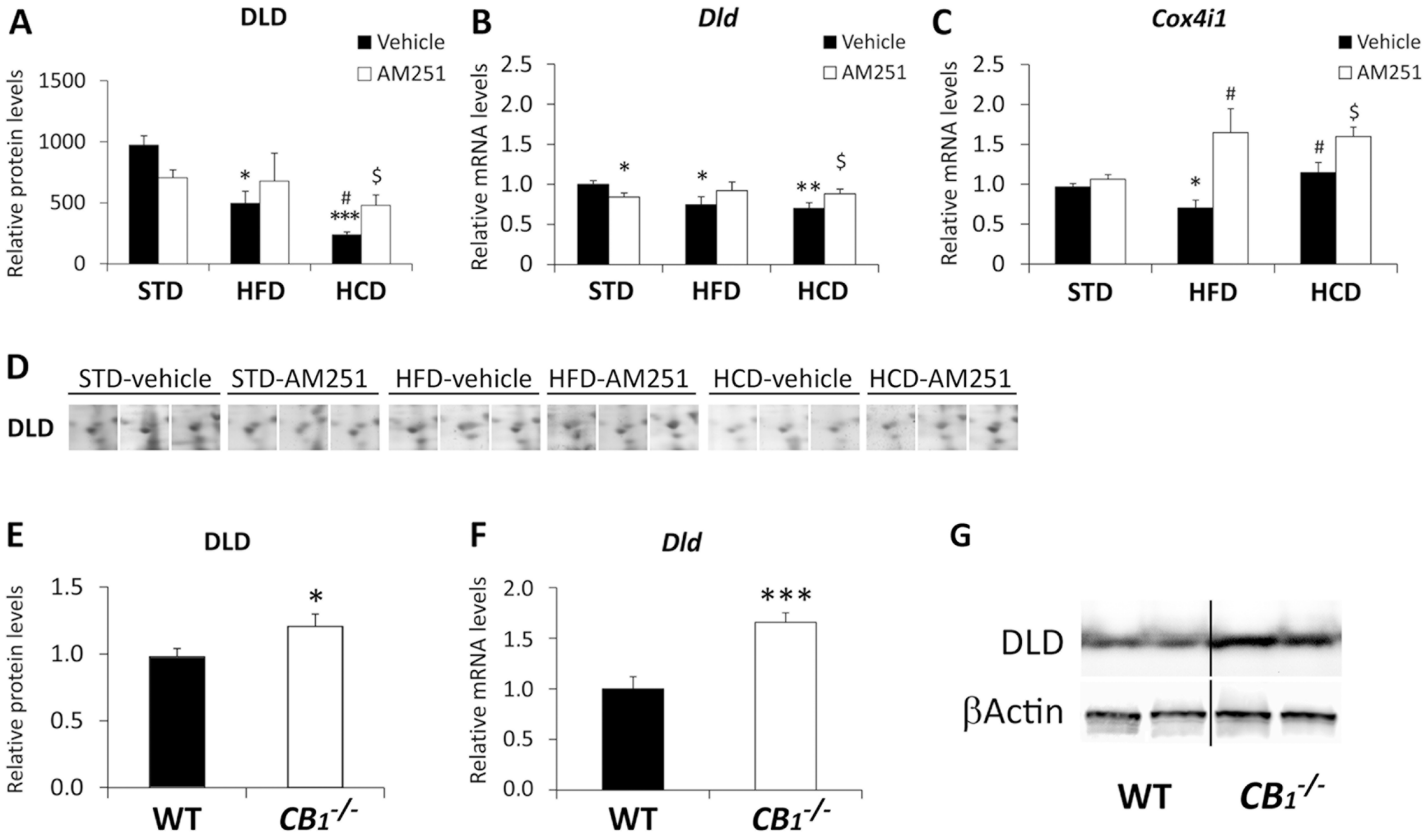
AM251 regulates the expression of DLD and Cox4i1. AM251 effects on the protein and gene expressions of DLD (**A**,**B**) and the gene expression of *Cox4i1* (**C**) in the abdominal muscle of STD, HFD and HCD-fed rats. Representative 2D polyacrylamide gels showing the intensity of the identified spots for DLD (**D**). Bonferroni’s test (*n* = 6–8): *P<0.05, **P<0.01, ***P<0.001 *vs*. STD-vehicle; ^#^ *P*<0.05 *vs*. HFD-vehicle; ^$^ *P*<0.05 *vs*. HCD-vehicle. Gene (**D**) and protein (**E**) expressions of DLD in the abdominal muscle of wild-type and *CB*_*1*_^*-/-*^ mice. Representative immunoblots (**F**). Student’s *t* test (*n* = 6): *P<0.05, ***P<0.001 *vs*. WT.

We analyzed the muscle expression of DLD enzyme, an E3 component of the mitochondrial pyruvate/α-ketoglutarate dehydrogenase complexes, which regulates TCA cycle activity and cellular respiration (Fig 6). Two-way ANOVA showed a treatment effect on the DLD protein expression (*F*_2,30_ = 8.47, *P* = 0.0012). Interaction in *Dld* expression (*F*_2,37_ = 3.24, *P* = 0.05) indicated that AM251 differentially modified its expression in a diet-dependent manner. Regarding Bonferroni test, both HFD and HCD decreased the protein and gene expressions of DLD (*^/^**^/^***P<0.05/0.01/0.001) (Fig 6A and 6B). Decreased protein expression of DLD in HCD-fed rats compared with HFD ones (^#^ *P*<0.05) was also observed. Increased protein and gene expressions of DLD was specifically detected in the muscle of AM251-treated HCD-fed rats (^$^ *P*<0.05) (Fig 6A and 6B) and confirmed in *CB*_*1*_^*-/-*^ mice (*^/^***P<0.05/0.001) (Fig 6D and 6E).

We also analyzed the gene expression of *Cox4i1*, a mitochondrial respiratory chain enzyme (Fig 6C). Two-way ANOVA showed a diet effect on *Cox4i1* expression (*F*_1,40_ = 15.66, *P* = 0.0003). The presence of interaction (*F*_1,40_ = 3.8, *P* = 0.03) indicated that AM251 differentially modified the *Cox4i1* expression in a diet-dependent manner. Thus, HFD induced a decreased *Cox4i1* expression when it was compared with STD and HCD (*^/#^ *P*<0.05). However, AM251 increased the *Cox4i1* expression in both HFD and HCD-fed rats (^#/$^ *P*<0.05) (Fig 6C).

### Localization of the CB_1_ receptors in the rodent striate muscle

**Fig 7:**
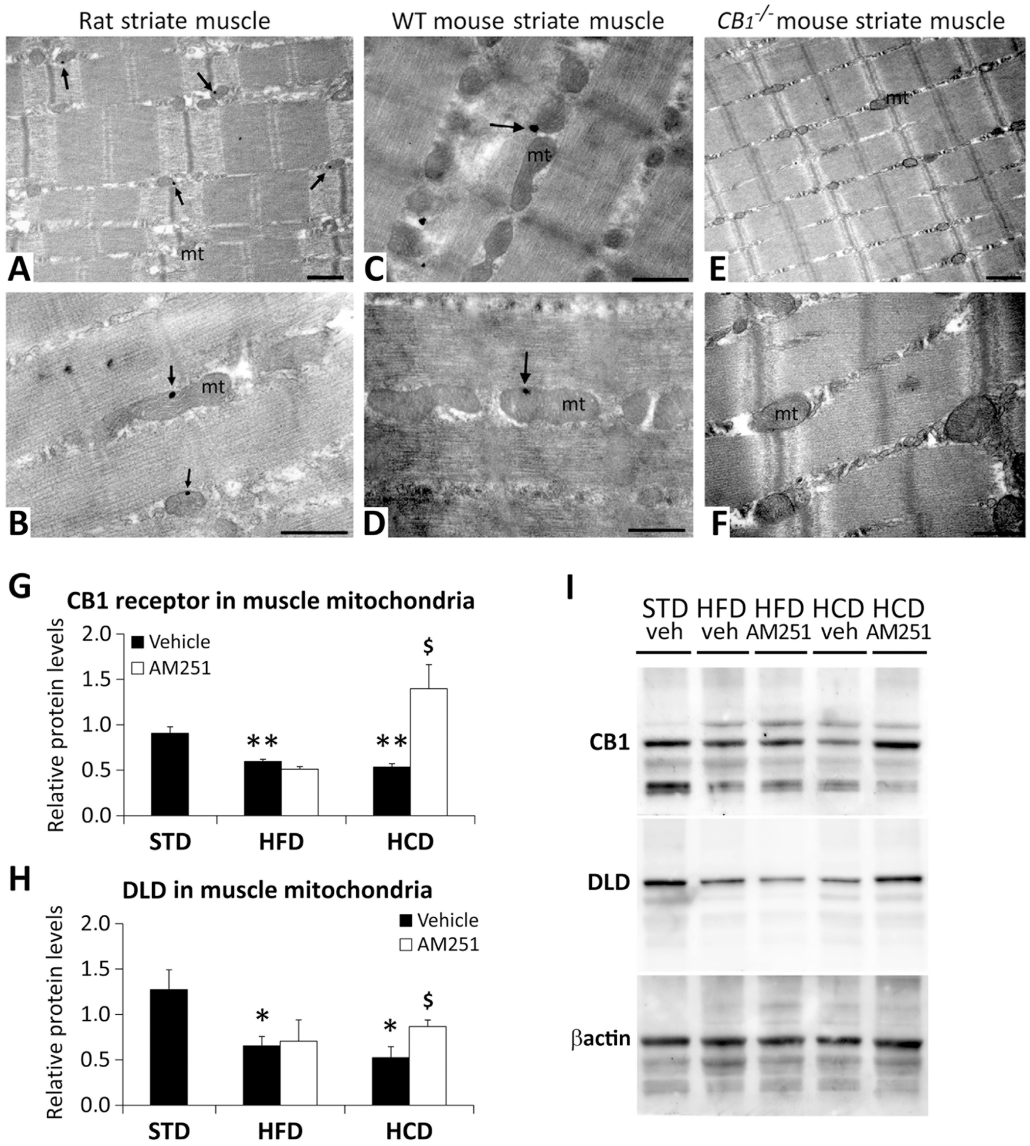
CB_1_ receptors in the striate muscle and AM251 effects on the mitochondrial CB_1_ receptor and DLD. Electron immunogold detection of CB_1_ receptors (arrows) on the mitochondrial (mt) membrane of striate muscle cells in rats (**A**,**B**), and wild-type (**C**,**D**) and *CB*_*1*_^*-/-*^ (**E**,**F**) mice. Scale bars: 0.5μm. AM251 effects on the protein expression of CB_1_ receptor (**G**) and DLD (**H**) in the purified mitochondrial fraction of the abdominal muscle of STD, HFD and HCD-fed rats. Representative immunoblots (**I**). Bonferroni’s test (*n* = 8): *P<0.05, **P<0.01 *vs*. STD-vehicle; ^$^ *P*<0.05 *vs*. HCD-vehicle.

Little is known concerning the expression and subcellular distribution of CB_1_ receptors in muscles. Therefore, we set to analyze CB_1_ receptor expression in the muscle abdominalis using immunogold electron microscopy (Fig 7). Interestingly, images showed immunogold particles labeling CB_1_ receptors in the outer membrane of the mitochondria of the rat and mouse striate muscle (Fig 7A–7D), whereas no electrodense particles were found in the striate muscle of *CB*_*1*_^-/-^ mouse (Fig 7E and 7F). Thus, the above-described effects of the CB_1_ receptor antagonist AM251 on glucose/pyruvate/lactate pathways might be partially mediated by direct modulation of mitochondrial functions.

### AM251 increases the protein expression of CB_1_ receptor and DLD in the muscle mitochondria of HCD-fed rats

To assess the above affirmation, the presence and protein expression of the CB_1_ receptor and DLD in the mitochondrial fraction of muscle of AM251-treated DIO rats were evaluated by Western immunoblotting (Fig 7G–7I). Two-way ANOVA showed diet and treatment effects, and interaction between factors on CB1 receptor expression in the muscle mitochondria (*F*_1,12_ = 8.20, *P* = 0.014; *F*_1,12_ = 9.22, *P* = 0.010; *F*_1,12_ = 12.18, *P* = 0.004 respectively). No significant effect on the DLD protein expression was observed. Regarding Bonferroni test, both HFD and HCD decreased the protein expression of CB_1_ receptor and DLD (*^/^**P<0.05/0.01) (Fig 7G and 7H). However, AM251 specifically increased the protein expression of CB_1_ receptor and DLD in the muscle mitochondria of HCD-fed rats (^$^ *P*<0.05) (Fig 7G and 7H).

### AM251 increased diaphorase/oxidative activity in the C_2_C_12_ cell mitochondria

**Fig 8:**
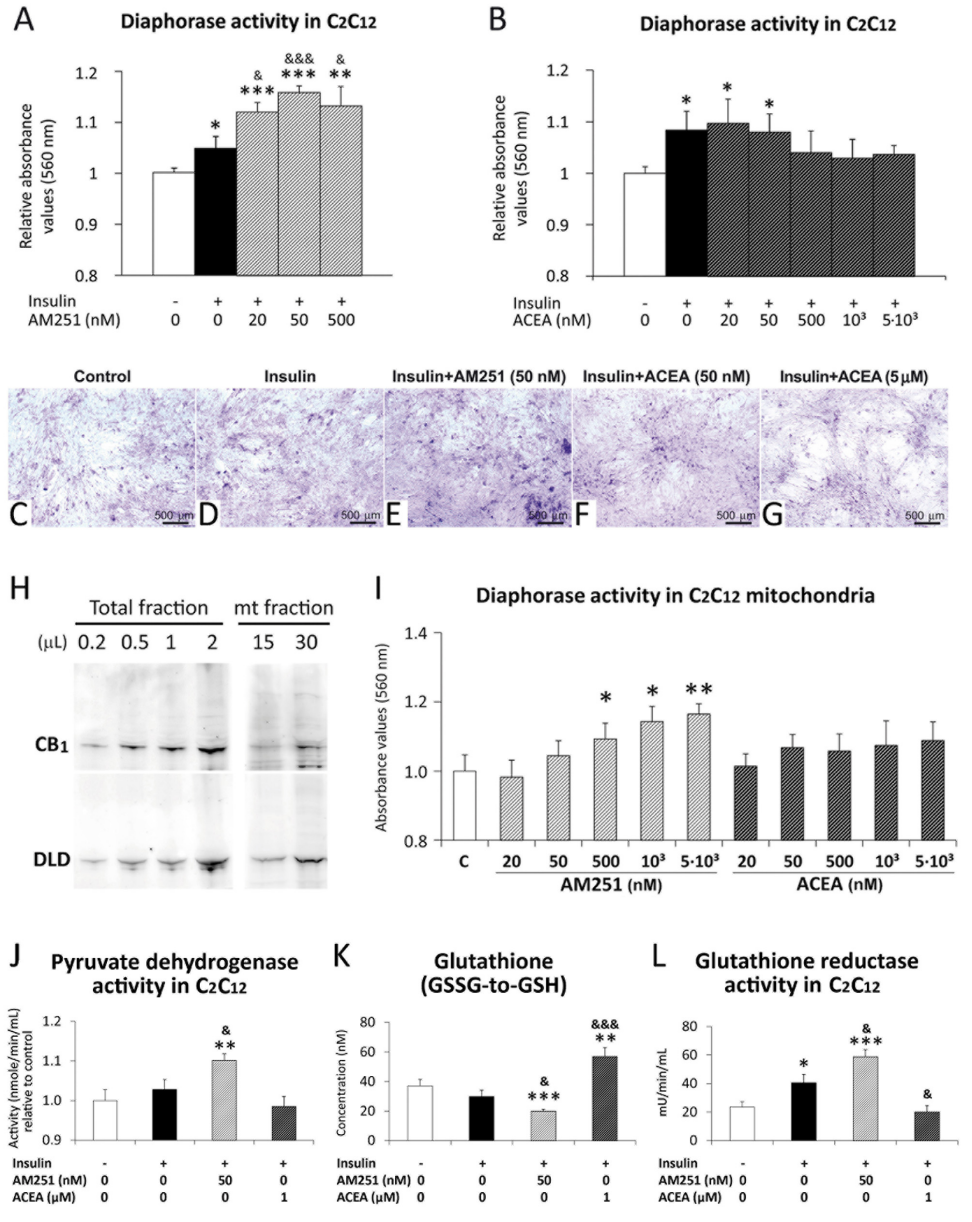
AM251 increased diaphorase/oxidative, pyruvate dehydrogenase and glutathione reductase activity. AM251 and ACEA effects on the diaphorase/oxidative activity in C_2_C_12_ myotubes in culture (**A**,**B**), and in the C_2_C_12_ mitochondrial fraction *in vitro* (**I**). AM251 and Representative images of differentiated C_2_C_12_ cells showing NBT staining (**C**-**G**). Representative immunoblots of CB_1_ receptor and DLD from the total and mitochondrial protein fractions of C_2_C_12_ myotubes (**H**). ACEA effects on the pyruvate dehydrogenase, glutathione content and glutathione reductase activity in C_2_C_12_ myotubes in culture (**J-L**). ANOVA (*n* = 8): *P<0.05, **P<0.01, ***P<0.001 *vs*. control C_2_C_12_ (white bar); ^&^ *P*<0.05, ^&&&^ *P*<0.001 *vs*. insulin C_2_C_12_ (black bar).

Given that the diaphorase/oxidative activity of the DLD enzymes plays a critical role through redox reactions in the TCA cycle, we aimed at clarifying the biological significance of the increased DLD expression in the muscle mitochondria after CB_1_ receptor pharmacological blockade. To this end, we first investigated the effect of the CB_1_ receptor blockade (AM251) and activation (ACEA) on the diaphorase activity of C_2_C_12_ myotubes in culture (Fig 8A–8G). Under conditions of basal oxidative state, insulin induced an increase of diaphorase/oxidative activity (*P<0.05) (Fig 8A and 8B). AM251 produced an enhancement of this effect when it was compared with either control group (**^/^***P<0.01/0.001) or insulin group (^&/&&&^ *P*<0.05/0.001) (Fig 8A and 8C–8E). Moreover, AM251 at a dose of 50 nM showed a maximal increase of the diaphorase/oxidative activity (Fig 8A and 8E). However, no specific effect or an opposed effect on the diaphorase/oxidative activity were observed when the C_2_C_12_ myotubes were treated with increased concentrations of ACEA (Fig 8B, 8F and 8G).

After checking the protein expression of CB_1_ receptor and DLD in the mitochondrial fraction of C_2_C_12_ myotubes (Fig 8H), we then investigated the effect of the CB_1_ receptor blockade (AM251) and activation (ACEA) on the diaphorase activity of C_2_C_12_ myotube mitochondria *in vitro* (Fig 8I). AM251 produced a dose-response increase of diaphorase/oxidative activity when it was compared with the control group (*^/^**P<0.05/0.01). However, no effect was observed when the mitochondrial fractions of C_2_C_12_ myotubes were incubated with ACEA (Fig 8I).

### AM251 increased pyruvate dihydrogenase and glutathione reductase activity in the C_2_C_12_ myotubes

To further assess the increased diaphorase activity and the putative mitochondrial ROS production in the C_2_C_12_ myotubes after CB_1_ receptor pharmacological blockade, we evaluated the pyruvate dehydrogenase and glutathione reductase activity (Fig 8J–8L). Under conditions of basal oxidative state, insulin induced an increase of glutathione reductase activity (*P<0.05) (Fig 8L). AM251 at 50 nM induced an enhancement of pyruvate dehydrogenase and glutathione reductase activity, and showed a lower GSSG-to-GSH rate (as a consequence of a low GSSG content), when it was compared with either control group (**^/^***P<0.01/0.001) or insulin group (^&^ *P*<0.05) (Fig 8J–8L). In contrast, ACEA at 1μM produced an increased GSSG-to-GSH rate (elevated GSSG content) and a decreased activity of glutathione reductase, when it was compared with either control group (**P<0.01) or insulin group (^&/&&&^ *P*<0.05/0.001) (Fig 8K and 8L).

## Discussion

**Fig 9:**
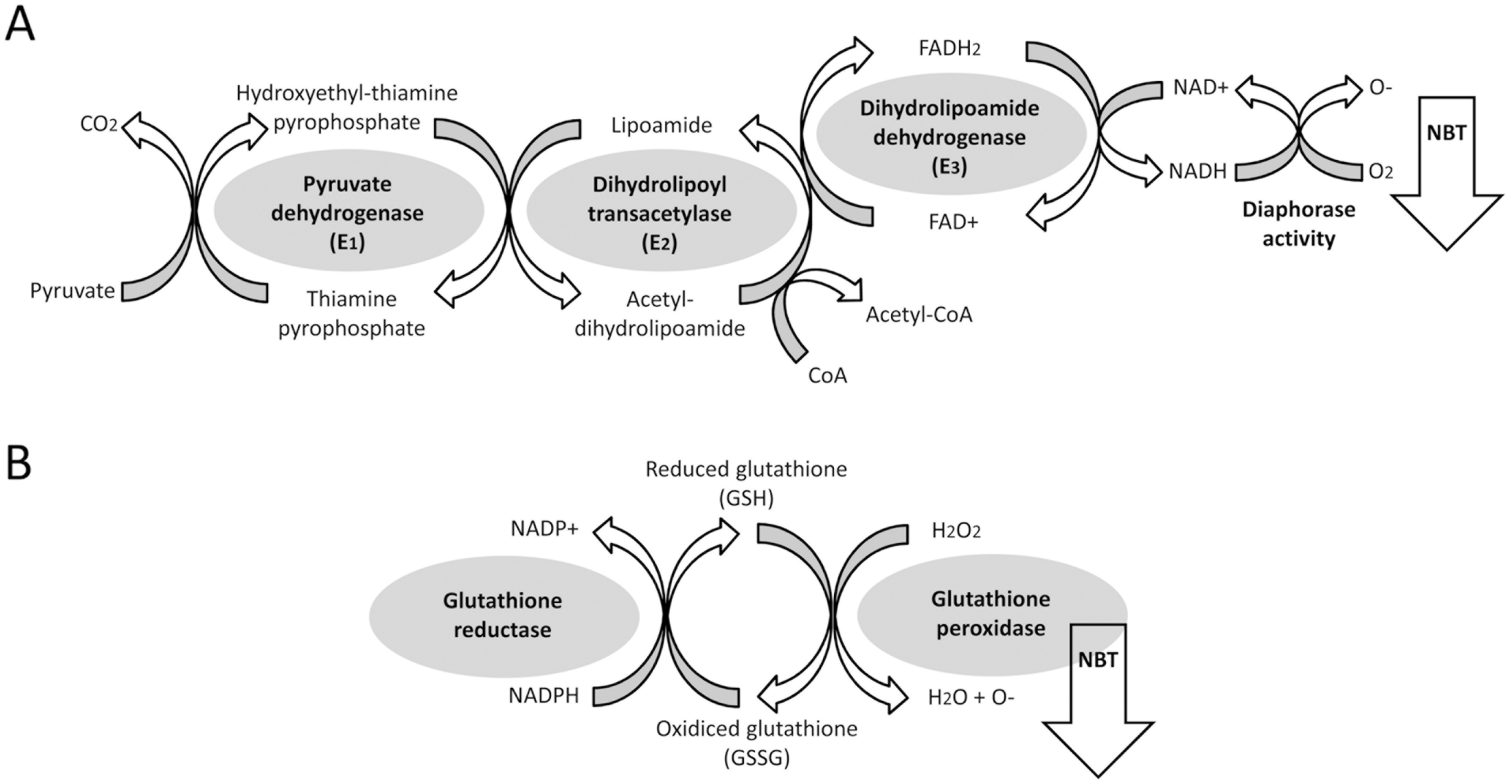
Scheme showing the enzymatic reactions that catalyze the pyruvate dehydrogenase complex, including pyruvate dehydrogenase activity and diaphorase activity of DLD (A) as well as the glutathione reductase/peroxidase activity (B). NBT staining is proportional to O^-^ production from redox activity.

Here we demonstrated that the expression of relevant metabolic enzymes involved in the regulation of glucose (GPI, TPI), pyruvate (Eno3, PKM1, LDHa), glyoxalase-1 (Glo1) and TCA and amino acid catabolism (DLD) pathways in the abdominal rat muscle was altered in a hypercaloric diet-dependent manner. The significant alteration in the protein expression of GPI, TPI, Eno3, LDHa, Glo1 and DLD showed a complete normalization after the systemic administration of the CB_1_ receptor antagonist AM251 at the effective dose of 3 mg kg^-1^ for 14 days in the obese rats specifically fed with a HCD. AM251 effects on muscle metabolism should be associated with a reduction in food/caloric intake and body weight gain [5,6,10]. Firstly, we observed that AM251 amended the HCD-induced increase of the protein expression of GPI, TPI, Eno3 and LDHa, and alleviated the HCD-induced decrease of the protein expression of Glo1. Secondly, we described that both CB_1_ receptor antagonism and deletion (*CB*_*1*_^*-/-*^) specifically displayed similar changes in the expression of the muscle DLD, an E3 component of the mitochondrial pyruvate/α-ketoglutarate/branched-chain keto acid (BCKDC) dehydrogenase complexes with dehydrogenase/diaphorase activity (Fig 9A). Thus, AM251 counteracted the HCD-induced decrease in the protein and gene expression of DLD in rat muscle, an effect that was confirmed after the protein analysis of DLD in the muscle mitochondrial fraction. Thirdly, we identified the presence of CB_1_ receptors at the outer membrane of striate muscle mitochondria, as was previously described in brain, suggesting a direct endocannabinoid regulation of mitochondrial activity in the muscle [31]. Moreover, we showed that AM251 reversed the HCD-induced decrease in the protein expression of CB_1_ receptor in the muscle mitochondrial fraction. Finally, regarding these results and the relevance of the redox activity of DLD in the TCA cycle, we evaluated the influence of the CB_1_ receptor blockade (AM251) and activation (ACEA) in the dehydrogenase and diaphorase/oxidative activity that can be detected *in vitro* in a cell model of differentiated C_2_C_12_ myotubes. We observed that AM251, but not ACEA, induced an enhancement of the diaphorase activity detected in the mitochondria of myotube- differentiated C_2_C_12_ cells, which was in accordance with the increased activity of pyruvate dehydrogenase and glutathione reductase in the C_2_C_12_ myotubes (Fig 9).

These results agree with the fact that CB_1_ receptor antagonism has a direct effect on peripheral energy utilization [4]. Although the ECS has been shown to be expressed in the skeletal muscle [5,7], little information about the effects of CB_1_ receptor blockade on muscle oxidative pathways and energy expenditure have been reported [8]. The present study demonstrated the ability of the CB_1_ receptor antagonist AM251 to restore the expression levels of specific enzymes, such as GPI, TPI, Eno3 and LDHa, related with glucose and pyruvate metabolism in an insulin-highly sensitive tissue (striate muscle), which have been specifically increased after the chronic administration of a high-carbohydrate diet. We interpreted that the up-regulation of the glucose/pyruvate metabolism enzymes can be a response to catalyze the excess of glucose availability. Consequently, the down-regulation of these metabolic enzymes by AM251 under a highly-carbohydrate context can be contributing to the improved glycaemia and insulin resistance described in previous studies [19,32]. Both mitochondrial respiratory activity and biogenesis were decreased in the skeletal muscle of obese/diabetic animal models and humans [33,34]. Thus, CB_1_ receptor activation impaired mitochondrial biogenesis in peripheral tissues, whereas the blockade showed opposite effects [35,36]. Because the cannabinoid receptor blockade has been implicated in the regulation of mitochondrial function, we focused in the mitochondrial candidate identified by the proteomic approach: the DLD, a relevant TCA cycle enzyme with NADH-related diaphorase activity coupled to mitochondrial respiration. We demonstrated that both HFD and HCD decreased the mitochondrial DLD expression in muscle. However, the abdominal muscle of HFD-fed rats, but not HCD-fed rats, showed a decreased expression of the mitochondrial respiratory chain gene *Cox4i1*. These results can be interpreted as the mitochondrial bioenergetics is particularly impaired after an excess of fatty acid availability by the diet, but seems less relevant in a highly-carbohydrate context [34]. In contrast, AM251 increased the *Cox4i1* gene expression in both HFD and HCD-fed rats, but it only increased the mitochondrial DLD expression in HCD-fed rats. In this case, the net effect of AM251-induced blockade of CB1 receptor in muscle might be an activation of mitochondrial respiration and, as a consequence, a potential increase in energy expenditure under a highly-carbohydrate context. Nonetheless, these potential effects on mitochondrial respiration need to be confirmed in future experiments.

DLD and *Cox41i* up-regulation by AM251 must be coupled to the increased diaphorase/oxidative, pyruvate dehydrogenase and gluthatione reductase activity specifically detected in C_2_C_12_ myotubes under an elevated availability of glucose (25 mM) in the differentiation culture medium. We propose that a higher production of NADH by the pyruvate dehydroganase activity and the increased diaphorase/oxidative activity, probably partaken by DLD, can be a response to the increased acidification of the mitochondrial matrix [37]. In our study, a higher level of protons in the mitochondrial matrix can be deduced from the up-regulation of the mitochondrial respiratory chain gene *Cox41i*. It has been described that the skeletal insulin resistance and the onset of type 2 diabetes produced an impairment of pyruvate dehydrogenase activity, which is, in turn, potently activated by exercise [38]. Our results suggest that the CB1 receptor antagonist AM251 could also counteract this deleterious effect on pyruvate dehydrogenase activity. We detected an increased activity of glutathione reductase after AM251 treatment. Because this enzyme is a key cellular antioxidant, our results suggest that the CB_1_ receptor inhibition can boost the breakdown of dangerous reactive molecules such as hydrogen peroxide [39,40].

Until recently, the effects of cannabinoids on oxidative metabolism were interpreted either as an indirect activation of plasma membrane CB_1_ receptors, or as unspecific alterations of the mitochondrial membranes [41]. However, novelty studies [31,42] challenged this concept as CB_1_ receptors are also present in the mouse neuronal mitochondria and can directly modulate neuronal energy metabolism. For instance, different CB_1_ receptor agonists (THC, WIN55,212–2, HU210) couldn’t decrease respiration rates of purified brain mitochondria from *CB*_*1*_^-/-^ mice [31]. We demonstrated that CB_1_ receptors are also present in the muscle mitochondria by using immunogold electron microscopy. As a consequence, in agreement with previous studies, we suggest that CB_1_ receptor might directly regulate mitochondrial metabolism in muscle by targeting the pyruvate dehydrogenase activity and the diaphorase/oxidative activity, probably partaken by DLD [43,44]. The confirmation of this hypothesis will be addressed in future studies.

In conclusion, we evidenced that the pharmacological blockade of CB_1_ receptors by AM251 at an effective dose of 3 mg kg^-1^ preferably modulates the muscle expression of selected glucose/pyruvate metabolic enzymes and the mitochondrial TCA cycle (and amino acid oxidation) enzyme DLD, which can promote an increased diaphorase/oxidative activity in response to an excess of carbohydrate availability. These results coupled to the presence of CB_1_ receptors in the muscle mitochondria demand further studies in order to support a new mechanism of action on energy expenditure through the muscle mitochondrial metabolism.

## Supporting Information

S1 Text Extended methodology. (PDF)

## Abbreviations

ACEA: arachidonyl-2’-chloroethylamide
Actb: β-actin
CB_1_: cannabinoid receptor type 1
Cox4i1: cytochrome c oxidase subunit 4 isoform 1, mitochondrial
DLD: dihydrolipoamide dehydrogenase
ECS: endocannabinoid system
Eno3: β-enolase
GADPH: glyceraldehyde 3-phosphate dehydrogenase
Glo1: glyoxilase 1
GPI: glucose-6-phosphate isomerase
GSH: reduced glutathione
GSSG: oxidized glutathione
Gusb: β-glucuronidase
HCD: high carbohydrate diet
HFD: high fat diet
HPLC-ESI MS: high-performance liquid chromatography-electrospray ionization mass spectrometry
LDHa: lactate dehydrogenase
MALDI-TOF MS: matrix-assisted laser desorption/ionization time-of-flight mass spectrometry
NADPH: nicotinamide adenine dinucleotide phosphate
NBT: nitroblue tetrazolium
PKM: pyruvate kinase
STD: standard diet
TPI: triosephosphate isomerase 1
