# Supplementary material for: Pharmacological Blockade of Cannabinoid CB1 Receptors in Diet-Induced Obesity Regulates Mitochondrial Dihydrolipoamide Dehydrogenase in Muscle
Source: PLoS One. 2015 Dec 15;10(12):e0145244. doi: 10.1371/journal.pone.0145244 (PMC4682857; doi:10.1371/journal.pone.0145244)
Supplement: S1 Text — (PDF) [file pone.0145244.s001.pdf]

SUPPLEMENTARY INFORMATION

EXTENDED MATERIALS AND METHODS

Ethics statements

All experimental procedures with animals were carried out in strict accordance with the recommendations in the European Communities directive 2010/63/EU and Spanish legislation (Real Decreto 53/2013, BOE 34/11370-11421, 2013) regulating the care and use of laboratory animals. The protocol was approved by the Ethics Committee for Animal Experiments of the University of Malaga (Permit number: 2014/0003). All efforts were made to minimize animal suffering and to reduce the number of animals used.

Animals

Ten-weeks-old male Wistar rats were used (Charles Rivers, Barcelona, Spain). Seven-weeks-old male $CB_1^{-/-}$ mice on a C57BL/6NCrl background were obtained, bred and genotyped as described (Marsicano et al., 2002). Animals were housed in pairs in cages maintained in standardized conditions of animal facilities at 20±2ºC room temperature, 40±5% relative humidity and a 12-hours light/dark cycle (on at 8 am and off at 8 pm) with dawn/dusk effect. Water and food were available *ad libitum* throughout the course of the study.

Diet-induced obesity (DIO)

Rats (*n* = 16 per group) were fed *ad libitum* for 12 weeks with three different types of diet: a regular chow diet (standard diet, STD) purchased from Harlam Teklad, Madison WI, a high-fat diet (HFD, 60% fat diet; D12492), or a high-carbohydrate diet (HCD, 70% carbohydrate diet; D12450B) both purchased from Research Diets Inc. (New Brunswick, NJ, USA). The HFD and HCD were used in order to induce obesity (Vida et al., 2014). The STD contains 2.9 kcal g^{-1} (6% fat, 20% protein), the HFD diet consisted of 5.24 kcal g^{-1} (of which, 20% of the

metabolizable energy content was protein, 20% carbohydrates and 60% fat) and the HCD had 3.85 kcal g^{-1} (with 20% protein, 70% carbohydrates and 10% fat). The HFD contained fat constituted by soybean oil (9.26 kcal% of total fat content) and lard (90.74 kcal% of total fat content) while the HCD contained carbohydrates constituted by corn starch (45 kcal% of total carbohydrate content), maltodextrin (5 kcal% of total carbohydrate content) and sucrose (50 kcal% of total carbohydrate content).

AM251 treatment

A dose-response study was firstly tested to select the most effective treatment for the repeated study. Rats (*n*=8) fed with STD received one intraperitoneal (i.p.) injection of either vehicle (1 mL kg^{-1} of 10% Tocrisolve in saline) or CB_1 receptor antagonist AM251 [N-(piperidin-1-yl)-5-(4-iodophenyl)-1-(2,4dichlorophenyl-4-methyl-1H-pyrazole-3-carboxamide] (Tocris) at doses of 0.3, 1, 3 and 10 mg kg^{-1} of body weight. The cumulative food intake was measured over a time course of 30, 60, 120 and 240 minutes in rats previously food-deprived for 24 hours with *ad libitum* access to water. The minimal dose at which treatment showed a robust effect on food intake was selected for the repeated treatment experiment (Fig. S1A).

For repeated treatment, rats (*n*=8) fed with STD and HFD for 10 weeks received a daily intraperitoneal injection of vehicle or AM251 at a dose of 3 mg kg^{-1} over 14 days (Fig. S1B). Food intake and body weight were monitored every two days along feeding (Fig. S1C-E) and treatment (Fig. S1F-H). We generated six experimental groups (*n*=8): STD-vehicle, STD-AM251, HFD-vehicle, HFD-AM251, HCD-vehicle and HCD-AM251.

Sample collection

Free-feeding rats from the six experimental groups were anaesthetized (sodium pentobarbital, 50 mg kg^{-1}, i.p.) two hours after the last dose of treatment in a room separate from the other

experimental animals. Striate muscle from the abdominal wall was dissected, immediately frozen in liquid nitrogen and kept at -80 ºC until mRNA or protein extraction for subsequent analyses. The muscles collected from rats were also prepared for histological examination. Samples were cut into small pieces, immediately fixed in 4% paraformaldehyde for 24 hours, and embedded in paraffin. Then samples were cut by microtome (5-µm thick), mounted on D-polylysinated glass slides, deparaffinized in xylene, and stained with haematoxylin and eosin for the evaluation of muscle fiber size, histological structure and inflammatory state by using an optical microscope (Olympus) (see Figure S1H-M). Muscle samples from the abdominal wall of the $CB_1^{-/-}$ and wild-type mice (n = 6) followed the same procedure described above.

Preparation of soluble protein fraction

Samples of rat abdominal muscle were placed on a homogenization buffer (10 mM HEPES, 10 mM NaCl, 1 mM K_2HPO_4, 5 mM $NaHCO_3$, 5 mM EDTA Na_2x2H_2O, 1 mM $CaCl_2x2H_2O$, 0.5 mM $MgCl_2x6H_2O$) supplemented with a protease inhibitor cocktail (Roche Complete tablets). Then, 100 µl of 2.5 M sucrose was added and centrifuged at 6,400 *g* for 5 minutes. The supernatant was placed into a new tube and centrifuged at 40,000 *g* for 45 minutes at 4° C. Protein content of the supernatant, correspondent to the cytoplasm fraction of samples, was measured using the Bradford assay. The supernatant was placed into a new tube and frozen at -20° C until the two-dimensional (2D) electrophoresis analysis.

2D gel electrophoresis

Triplicate 2D polyacrylamide gels were performed for each animal to minimize the effects of intra-assay variation. Isoelectric focusing (IEF) was performed with a Protean IEF cell (BioRad), using IPG (immobilized pH gradient) gel strips pH 3-10 NL 11 cm long. Equivalent amounts of protein extract (30 µg) were added to rehydration buffer (8M urea, 2

M thiourea; 4% CHAPS; 0,4% IPG buffer pH 3-10, 0,002% bromophenol blue, 40 mM DTT) until a final volume of 200 µl, and added to the strips. Gel strips were rehydrated for 18 hours, at 50 V and 20° C, and focused at 40 kV h^{-1} at 20° C. Then, the strips were incubated in two equilibration buffer (1.5 M Tris-HCl pH 8.8, 6 M urea, 30% glycerol, 2% SDS), first with 130 mM dithiothreitol and after with 135 mM iodoacetamide. The separation of protein in the second dimension was performed on Criterion XT Bis-Tris 12% polyacrylamide gel at 80 V for 20 minutes and then at 160 V for 70 minutes at room temperature. The 2D gels were fixed for 16 hours in a fix solution (40% ethanol, 10% acetic acid) and stained using a method compatible with MS (Dodeca™ silver stain kit, Bio-Rad). The stained gels were analyzed using PDQuest® Software v7.1 (Bio-Rad). Normalization of spots was made to compare the densities of the same spot (amount of protein) on other gels by using the total density in gel image. Data were expressed as parts per million (PPM) of the summed OD value of all pixels within the image. This model assumes that all information captured in an image from both background and the spots will be relatively consistent from gel to gel.

MALDI-TOF mass spectrometric analysis

Spots with densities that significantly differed between diets and/or treatments were excised from the SDS-PAGE gels using the BioRad spot cutter. Gel plugs were directly placed into a 96-well V-bottomed plate with 100 µL of water that was removed immediately before the trypsination process. The proteins were trypsinized using the MassPrep Station (Waters, Micromass, Manchester, UK) protocol, which includes sequentially: destaining steps for silver removal, reduction of the protein with DTT, alkylation of the protein with iodoacetamide, removal of DTT and iodoacetamide, dehydration of the gel plug, incubation with trypsin, and extraction of the peptides. Of the extracted peptides, 1 µL was applied to the target area of a 96 × 2 teflon MALDI target plate (Applied Biosystems, Warrington, UK) and

allowed to dry to ~50% of the original volume. At this point, 0.5 μL of an α-cyano-4-hydroxycinnamic acid matrix solution (5 mg/ml in 70% acetonitrile/H_2O, 0.1% TFA) was applied to the target. The samples were dried in a stream of air before MALDI MS analysis.

MALDI–time of flight (TOF) MS was performed using an Applied Biosystems Voyager-DE PRO in reflectron mode. Each spectrum was obtained using 500 shots of the appropriate laser power and, where appropriate, spectra were accumulated and filed. A macro was applied, which allowed baseline correction and de-isotoping of the peptide mass peaks. A peptide mass list of the most intense peaks was generated automatically, and this list was pasted into Matrix Science Mascot by using the MSDB database during the search. We set the following search criteria: allowance of 0 or 1 missed cleavages, trypsin as digestion enzyme, carbamidomethyl modification of cysteine, methionine oxidation as partial modification, and charged state as MH^+ (De Roos et al., 2005).

Nano LC-ESI-CID-MSMS analysis

Gel plugs were also analyzed by nano-HPLC/ESI ion trap MS, using manual in-gel trypsin digestions of destained spots after reduction and alkylation with DTT and iodoacetamide, as described above. Gel pieces were washed, dehydrated and dried in the similar manner as previously described. Dried gel pieces were re-swollen and in-gel digested with 40 ng μl^{-1} trypsin (Promega Trypsin Gold, Madison, WI, USA) and incubated overnight at 30°C. Peptides were extracted with 100% acetonitrile + 0.1% formic acid.

LC-ESI-CID-MS/MS analysis was performed using an Agilent 1200 nano-HPLC system equipped with a Zorbax 300SB C-18 trap column (5 μm, 5 x 0,3 mm) and a Zorbax 300SB C-18 analytical column (3,5 μm, 150 mm x 75 μm). A HCT ultra ETD II (Bruker Daltonics, Bremen, Germany) was used to record peptide spectra over the mass range of m/z 350–1,500, and MS/MS spectra in information-dependent data acquisition over the mass range of m/z

100–2,800. MS/MS spectra were interpreted and peak lists were generated by Data Analysis 3.4 (Bruker). Searches were done by using the Mascot 2.2 (Matrix Science, London, UK) against latest NCBI and Swissprot database for protein identification. Searching parameters were set as follows: enzyme selected as trypsin with two maximum missing cleavage sites, species limited to rat, a mass tolerance of 0.2 Da for peptide tolerance, 0.5 Da for MS/MS tolerance, fixed modification of carbamidomethyl (C) and variable modification of oxidation (M). Repeatedly, MS spectra were recorded followed by three data-dependent CID MS/MS spectra generated from the three of highest intensity precursor ions. An active exclusion of 0.4 min after two spectra was used to detect low abundant peptides. The voltage between ion spray tip and spray shield was set to 1,500 V. Drying nitrogen gas was heated to 150 °C and the flow rate was 10 l min^{-1}. The collision energy was set automatically according to the mass and charge state of the peptides chosen for fragmentation. Multiple charged peptides were chosen for MS/MS experiments due to their good fragmentation characteristics (Bae et al., 2012).

RNA isolation and RT-PCR analysis

Samples of abdominal muscle tissue (~100 mg) were placed on 1 ml of Trizol® and then homogenized using a Tissuelyser II (Qiagen®). To ensure the purity of the mRNA sequences excluding molecules smaller than 200 nucleotides, RNA samples were isolated with RNeasy® Mini kit (Qiagen), which included digestion on a DNase I column (RNase-free DNase Set; Qiagen). Total mRNA concentration was quantified using a spectrophotometer (Nanodrop 1000 Spectrophotometer, Thermo Scientific, Rochester, NY) to ensure A260/A280 ratios of 2.0. Reverse transcription was carried out from 1 µg of mRNA using the Transcriptor Reverse Transcriptase kit and random hexamer primers (Transcriptor RT, Roche Diagnostic GmbH, Mannheim, Germany). Quantitative reverse transcription polymerase

chain reaction (quantitative RT-PCR) was performed using a C1000 Touch™ Thermal Cycler and the FAM dye label format for the TaqMan® Gene Expression Assays (Applied Biosystems). Each reaction was run in duplicate and contained 9 µl of cDNA diluted 1/100. Cycling parameters were: 50°C for 2 minutes to deactivate single- and double-strained DNA containing dUTPs, 95°C for 10 minutes to activate Taq DNA polymerase followed by 45 cycles at 95°C for 15 seconds for cDNA melting, and 60°C for 1 minute to allow for annealing and the extension of the primers, during which fluorescence was acquired. Melting curves analysis was performed to ensure that only a single product was amplified. We analyzed three housekeeping genes (*Actb*, *Gapdh* and *Gusb*) and selected the most suitable according to their homogeneity. Absolute values from each sample were normalized with regard to the housekeeping gene *Gapdh*. The relative quantification was calculated using the ΔΔCt method and normalized to the control group. Primers for the PCR reaction were obtained based on Applied Biosystems' genome database of rat and mouse mRNA references (http://bioinfo.appliedbiosystems.com/genome-database/gene-expression.html) (see Table S1) (Crespillo et al., 2011; Vida et al., 2014).

Protein extraction and Western blot analysis

Proteins from abdominal muscle of wild-type and $CB_1^{-/-}$ mice were extracted using RIPA buffer 1x (50 mM Tris HCl pH 7.4, 150 mM NaCl, 0.25% NaDOC, 1% Triton X-100, 1 mM EDTA pH 8) containing a protease and phosphatase inhibitor cocktail (5 mg ml^{-1} leupeptin, 100 mM NaF 5, 1 mM sodium orthovanadate, 5 µg ml^{-1} aprotinin, 1 µg ml^{-1} pepstatin A, 10 µg ml^{-1} trypsin inhibitor, 1 mM phenylmethylsulfonyl fluoride, 0.75 µl ml^{-1} protease and phosphatase inhibitor cocktail). The samples (~100 mg) were first incubated on the RIPA buffer 1x for 2 hours at 4°C with gentle agitation, then homogenized with an IKA-Ultra-Turrax® T8 homogenizer (IKA-Werke) and finally centrifuged at 20,800 *g* for 20 minutes at

4°C. The supernatants were collected into a new tube, diluted with loading buffer and frozen at -20° C. Protein concentration was determined by the Bradford protein assay. Equivalent amounts of protein extract (75 µg) were separated in gradient SDS-PAGE gels (Criterion XT Precast Gel with 3-8% polyacrylamide, Bio-Rad Laboratories, Inc.) and electroblotted onto nitrocellulose membranes (Bio-Rad). Membranes were blocked in TBS-T (50 mM Tris-HCl, pH 7.6; 200 mM NaCl and 0.1% Tween-20) with 2% albumin fraction V from bovine serum (BSA, Roche) at room temperature for 1 h. Specific proteins were detected by incubation in TBS-T and 2% BSA at 4ºC overnight with the corresponding primary antibodies (see Table S2). After extensive washings in TBS containing 1% Tween 20, a HRP-conjugated anti-rabbit IgG (H+L) or anti-mouse secondary antibodies (Promega) diluted 1:10,000 was added for 1 h at room temperature. After extensive washings in TBS containing 1% Tween 20, the specific protein bands were revealed using the enhanced chemiluminiscence detection system (Santa Cruz, Biotechnology Inc. CA, USA), in accordance with the manufacturer's instructions. Images were visualized in an Autochemi-UVP Bioimaging System. Bands were quantified by densitometric analysis performed by ImageJ software (Rasband, W.S., ImageJ, U.S., National Institutes of Health, Bethesda, MA, USA, http://imagej.nih.gov/ij, 1997-2012) Absolute values were normalized regarding β-actin levels (Crespillo et al., 2011; Vida et al., 2014).

Isolation of rat muscle mitochondria for protein (Western blot) analysis

The isolation of muscle mitochondria was realized as described previously (Benard et al., 2006) with modifications. Abdominal muscle was collected in isolation medium I (210 mM mannitol, 70 mM sucrose, 50 mM Trizma and 10 mM EDTA) and digested with trypsin (Gibco) at 0.5 mg g^{-1} for 30 minutes. Then, the reaction was stopped with a trypsin inhibitor and centrifuged at 1,000 *g* for 5 minutes. The supernatant was filtered and centrifuged at 7,000 *g* for 10 minutes. The obtained pellet was resuspended in an isolation medium II (225

mM mannitol, 75 mM sucrose, 10 mM Trizma and 0.1 mM EDTA) and centrifuged at 1,000 *g* for 5 minutes. The resulting supernatant was centrifuged at 7,000 *g* for 10 minutes. The obtained pellet (mitochondrial fraction) was resuspended in 50 µl of isolation medium II. Then, samples were homogenized by sonication and protein concentration was measured using the Bradford method with BSA as standard.

Western blotting of the mitochondrial protein extracts (30 µl) was performed as was described above. DLD and CB_1 proteins were detected by overnight incubation in the corresponding primary antibodies diluted 1:200 (Table S2). Absolute values were normalized regarding β-actin levels.

Cell culture and treatment

C_2C_{12} mouse C3H muscle myoblasts (cat. no. 91031101, Sigma-Aldrich) were cultured and differentiated as previously described (Iannotti et al., 2014). Proliferating C_2C_{12} cells were propagated in a growth medium composed of DMEM (ThermoFisher) supplemented with 5% fetal bovine serum (FBS), 20 mM HEPES, 100 U ml^{-1} penicillin and 100 µg ml^{-1} streptomycin, and 1% L-glutamine (ThermoFisher) in a humidified atmosphere of 95% air/5% CO_2 at 37ºC. Myotube differentiation was achieved upon exposure of proliferating C_2C_{12} cells to a differentiating medium (DM) containing DMEM (25 mM glucose) supplemented with 2% horse serum, 20 mM HEPES, 100 U ml^{-1} penicillin and 100 µg ml^{-1} streptomycin, and 1% L-glutamine for six days, according to the experimental needs. To assess C_2C_{12} myotube treatment, cells were first synchronized in fresh DM for 24 hours. Then, the cells were treated with the CB1 receptor agonist ACEA or the CB1 receptor antagonist AM251 at concentrations of 20, 50, 500, 10^3 and/or $5 \cdot 10^3$ nM for 2 hours. After treatment, the cells were first cultured in fresh DM without serum for 2 hours and then incubated in 10 nM insulin for 10 minutes. Finally, 0.2 mg ml^{-1} Nitroblue Tetrazolium (NBT)

was also added into the medium in order to activate and detect diaphorase/oxidative reaction. Then, the stained cells were disrupted and homogenised by sonication. The quantification of the staining was determined by optic density at 560 nm using a VersaMax Absorbance Microplate Reader (Molecular Devices).

Isolation of C_2C_{12} myotube mitochondria

Mitochondrial extraction from differentiated C_2C_{12} cells was realized as described previously (Benard et al., 2007) with modifications. Cells were trypsinised and centrifuged at 1,000 *g* for 10 minutes. The pellet was resuspended in a buffer I containing 210 mM mannitol, 70 mM sucrose, 5 mM HEPES, 1 mM EDTA and 0.5% BSA supplemented with 0.5 mM Tx100. After 15 minutes, permeabilization was verified using trypan blue. Samples were centrifuged at 625 *g* for 5 minutes and the pellet was resuspended in 10 ml of buffer I and homogenized with 60 gentle strikes in a glass potter. Membrane disruption was verified under the microscope, and cells were centrifuged at 625 *g* for 5 minutes. The supernatant was centrifuged at 10,000 *g* for 20 minutes, and the pellet (mitochondrial fraction) was resuspended in 100 µl of a respiration buffer (75 mM mannitol, 25 mM sucrose, 100 mM KCl, 10 mM Tris-HCl pH 7.4, 50 µM EDTA and 10 mM sodium pyruvate). Protein concentration was measured using the Bradford method with BSA as standard. Approximately, 1.1 mg of mitochondrial proteins per 10^6 cells was recovered.

Diaphorase activity measurement of C_2C_{12} mitochondrial fraction

Mitochondrial samples at a final protein concentration of 0.2 mg ml^{-1} were incubated in respiration buffer for 10 minutes at 37º C. Samples were first treated with AM251 or ACEA at 20, 50, 500, 10^3 and $5·10^3$ nM for 30 minutes. Then, NBT were added for an overnight

incubation at 37º C in order to detect diaphorase/oxidative reaction. Finally, samples were homogenized by sonication and measured at an optic density of 560 nm.

Preembedding immunogold method for electron microscopy

Muscular tissues were cut at 50 μm in a vibrotome and collected in 0.1M PB (pH 7.4) at room temperature (RT). Sections were preincubated in a blocking solution of 10% bovine serum albumin (BSA), 0.1% sodium azide and 0.02% saponin prepared in Tris-HCl buffered saline (TBS 1X, pH 7.4) for 30 minutes at RT. A preembedding silver-intensified immunogold method was used for the localization of CB_1 protein. Muscular sections were incubated in primary goat CB_1 polyclonal antibodies (2μg ml^{-1}; CB1-Go-Af450-1; Frontier Science Co. Ltd; 1-777-12, Shinko-nishi, Ishikari, Hokkaido, Japan) in 10% BSA/TBS containing 0.1% sodium azide and 0.004% saponin on a shaker for 2 days at 4°C.

After several washes in 1% BSA/TBS, tissue sections were incubated in a secondary 1.4 nm gold-labeled rabbit anti-goat IgG (Fab' fragment, 1:100, Nanoprobes Inc., Yaphank, NY, USA) in 1% BSA/TBS with 0.004% saponin on a shaker for 4 hours at room temperature. Thereafter, the tissue was washed in 1% BSA/TBS overnight at 4ºC and postfixed in 1% glutaraldehyde in TBS for 10 minutes at RT. Following washes in double-distilled water, gold particles were silver-intensified with a HQ Silver kit (Nanoprobes Inc., Yaphank, NY, USA) for about 12 minutes in the dark and then washed in 0.1M PB (pH 7.4). Stained sections were osmicated (1% OsO4 in 0.1M PB, pH 7.4, 20 minutes), dehydrated in graded alcohols to propylene oxide and plastic-embedded flat in Epon 812. 65nm ultrathin sections were collected on mesh nickel grids, stained with uranyl acetate and lead citrate, and examined in a Philips EM208S electron microscope. Tissue preparations were photographed by using a digital camera coupled to the electron microscope.

Semi-quantification of mitochondrial CB_1 in rat abdominal muscle

50-µm-thick muscular sections from each animal (*n*=3 each) showing good and reproducible silver-intensified gold particles were cut at 65 nm. Electron micrographs (10,000–25,000X) were taken from grids (132 µm side) containing silver-intensified gold particles; all of them showed a similar labeling intensity indicating that selected areas were at the same depth. Furthermore, to avoid false negatives, only ultrathin sections in the first 1.5 µm from the surface of the tissue block were examined. Positive labeling was considered if at least one immunogold particle was over mitochondria or within approximately 30 nm from the mitochondria membrane. Metal particles on mitochondrial membranes were visualized and counted. The numbers of labeled mitochondria were normalized to the total number of mitochondria in the images to identify the proportion of CB_1-positive mitochondria, as performed previously (Bénard et al., 2012; Hebert-Chatelain et al, 2014). Then, the percentage of immunolabeled mitochondria was calculated and displayed as mean ± S.E.M. Density of mitochondrial CB_1 immunolabeling was calculated as immuneparticles/µm membrane of positive mitochondria and shown as mean ± s.e.m. Image-J software (1.43u version, NIH, USA) was used to measure membrane length (see Figure S3).

Statistical analysis

All data for graphs were expressed as the mean ± standard error of the mean (s.e.m.) of at least 6-8 determinations per experimental group. Statistical results were obtained using the computer program GraphPad Prism version 5.04 (GraphPad Software Inc., San Diego, CA, USA). Statistical analysis was performed using two-way ANOVA with the two factors being diet (STD, HFD and HCD) and treatment (vehicle and AM251), followed by Bonferroni *post hoc* test for multiple comparisons, and one-tailed Student's *t* test with the *Welch* correction

applied when appropriate (no equal variances assumed). A *p*-value below 0.05 was considered statistically significant.

REFERENCES

Bae N, Yang JW, Sitte H, Pollak A, Marquez J, Lubec G. An electrophoretic approach to screen for glutamine deamidation. Anal. Biochem. 2012;428: 1-3.

Benard G, Bellance N, James D, Parrone P, Fernandez H, Letellier T, et al. Mitochondrial bioenergetics and structural network organization. J Cell Sci. 2007;120: 838-48.

Benard G, Faustin B, Passerieux E, Galinier A, Rocher C, Bellance N, et al. Physiological diversity of mitochondrial oxidative phosphorylation. Am J Physiol Cell Physiol. 2006;291: C1172-82.

Bénard G, Massa F, Puente N, Lourenço J, Bellocchio L, Soria-Gómez E, et al. Mitochondrial CB_1 receptors regulate neuronal energy metabolism. Nat Neurosci. 2012;15: 558-564.

de Roos B, Duivenvoorden I, Rucklidge G, Reid M, Ross K, Lamers RJ, et al. Response of apolipoprotein E*3-Leiden transgenic mice to dietary fatty acids: combining liver proteomics with physiological data. FASEB J. 2005;19: 813-5.

Hebert-Chatelain E, Reguero L, Puente N, Lutz B, Chaouloff F, Rossignol R, et al. Studying mitochondrial CB1 receptors: Yes we can. Mol Metab. 2014;3: 339.

Iannotti FA, Silvestri C, Mazzarella E, Martella A, Calvigioni D, Piscitelli F, et al. The endocannabinoid 2-AG controls skeletal muscle cell differentiation via CB1 receptor-dependent inhibition of Kv7 channels. Proc Natl Acad Sci USA. 2014;111: E2472-81.

Marsicano G, Wotjak CT, Azad SC, Bisogno T, Rammes G, Cascio MG, et al. The endogenous cannabinoid system controls extinction of aversive memories. Nature 2002;418: 530-534.

Vida M, Rivera P, Gavito AL, Suárez J, Pavón FJ, Arrabal S, et al. CB1 blockade potentiates down-regulation of lipogenic gene expression in perirenal adipose tissue in high carbohydrate diet-induced obesity. PLoS One 2014;9: e90016.
